# Agilik@home: A randomized controlled trial protocol to evaluate the effects of home-based training with the Agilik powered KAFO in children with cerebral palsy and crouch gait

**DOI:** 10.1371/journal.pone.0340877

**Published:** 2026-03-25

**Authors:** Roberta Nossa, Fabio Alexander Storm, Eleonora Diella, Riccardo Riboni, Luca Emanuele Molteni, Mattia Chiappini, Cristina Maghini, Elena Beretta, Roberta Nicotra, Sara Abbondio, Carolina Ferrante, Annalisa Cotardo, Giada Sgherri, Marco Germanotta, Maria Cristina Mauro, Alessio Fasano, Carlotta Cordoni, Maurizio Ferrarin, Anna Cavallini, Sabrina Giovanna Signorini, Giuseppina Sgandurra, Irene Aprile, Laura Iuvone, Angela Cavalagli, Emilia Biffi, on behalf of the Agilik@home Study Group

**Affiliations:** 1 Scientific Institute, IRCCS Eugenio Medea, Bosisio Parini (LC), Italy; 2 Department of Psychology, University of Torino, Torino, Italy; 3 Child Neuropsychiatry Unit, IRCCS Mondino Foundation, Pavia, Italy; 4 Department of Brain and Behavioural Sciences, University of Pavia, Pavia, Italy; 5 Department of Developmental Neuroscience, IRCCS Fondazione Stella Maris, Pisa, Italy; 6 Ph.D. Programme in Clinical and Translational Sciences, University of Pisa, Pisa, Italy; 7 IRCCS Fondazione Don Carlo Gnocchi, Firenze, Italy; 8 IRCCS Fondazione Don Carlo Gnocchi, Milano, Italy; 9 Department of Clinical and Experimental Medicine, University of Pisa, Pisa, Italy; Aichi Prefectural Mikawa Aoitori Medical and Rehabilitation Center for Developmental Disabilities, JAPAN

## Abstract

**Background:**

Cerebral palsy (CP) is the most common neuromotor disability in children and often results in spastic diplegia, which can lead to crouch gait. This gait pattern, resulting from weakness predominantly in the extensor muscles and spasticity mainly affecting the flexors, is characterized by excessive knee flexion, insufficient hip extension, and excessive ankle dorsiflexion, which together can progressively impair walking. The Agilik powered knee-ankle-foot orthosis is designed to enhance knee extension and potentially improve gait in children with CP. Here we present the protocol of a study that aims to evaluate the clinical effectiveness of Agilik in a domiciliary setting.

**Methods:**

According to this protocol, the multicentre randomized controlled trial will enrol 40 children with CP (aged 5–17 years) exhibiting crouch gait. Participants will be randomly assigned to either the Intervention Group, which will receive training with the Agilik device, or the Control Group, which will maintain their standard routines and therapies. All participants will undergo physical examination and baseline assessments. Then, while the Control Group continues the usual care, the Intervention Group will begin the Agilik program, including three device fitting visits, 4/5 weeks of hospital training, and two months of home-based sessions. Both groups will be evaluated at three time points: baseline, after the intervention, and at a one-month follow-up. Primary endpoints include the 6-Minute Walking Test and Knee Extension in Mid-Stance computed during gait analysis. Secondary endpoints assess gross-motor abilities, gait speed, 3D gait analysis with electromyography, joint range of motion, muscle length, self-perceived performance, spasticity, and balance. Additionally, the Intervention Group will provide feedback on the usability and acceptability of the device, and on cognitive, behavioural and affective engagement.

**Discussion:**

This trial will provide valuable insights into the effectiveness of the Agilik device in children with CP and crouch gait. It will help emphasizing the potential benefits of the device on daily living activities and gait performance. The findings could influence clinical practice and guide future interventions for managing crouch gait in CP, potentially leading to enhanced quality of life for these children.

**Trial registration:**

The trial has been registered the 30^th^ September 2024 on ClinicalTrials.gov with the identifier NCT06622655.

## Introduction

### Administrative information

**Table:** 

Title {1a}	Agilik@home: a randomized controlled trial protocol to evaluate the effects of home-based training with the Agilik powered KAFO in children with cerebral palsy and crouch gait
Trial registration {2a and 2b}.	The trial has been registered on ClinicalTrials.gov with the identifier NCT06622655.
Protocol version {3}	Version 1.0, dated April 20, 2024
Author details {5a}	IRCCS E. Medea – Associazione “La Nostra Famiglia” Via Don Luigi Monza, 20 23842 Bosisio Parini (LC) – Italy Tel. + 39 031 877.111, Fax + 39 031 877.499 IRCCS Fondazione Stella Maris Viale del Tirreno 341/ ABC 56128 Calambrone (PI) – Italia Tel. 050 886284 IRCCS Fondazione Mondino Via Mondino, 2 27100 Pavia (PV) – Italy Fondazione Don Carlo Gnocchi Onlus Via Maresciallo Caviglia, 30 00194 Roma (RM) – Italy Fondazione Don Carlo Gnocchi Onlus Via Casal del Marmo, 401 00166 Roma (RM) – Italy Fondazione Don Carlo Gnocchi Onlus – Centro S. Maria al Mare Via Leucosia, 16 84131 Salerno (SA) – Italy
	Fondazione Don Carlo Gnocchi Onlus – IRCCS S. Maria Nascente Via Alfonso Capecelatro, 66 20148 Milano (MI) – Italy
Name and contact information for the trial sponsor {5b}	Emilia Biffi, PhD, Biomedical Engineer Via Don Luigi Monza, 20 23842 Bosisio Parini (LC) – Italy Tel. + 39 031 877.862 Email: emilia.biffi@lanostrafamiglia.it Cristina Maghini, MD Via Don Luigi Monza, 20 23842 Bosisio Parini (LC) – Italy Tel. + 39 031 877.862 Email: cristina.maghini@lanostrafamiglia.it
Role of sponsor {5c}	In this study, the sponsor, who is also one of the study investigators, is responsible for the study design, data collection, management, analysis, interpretation, and the writing of the report. The sponsor will have ultimate authority over these activities. In contrast, the funder does not have a role in these aspects of the study. The decision to submit the report for publication will be made by the study team, ensuring that the sponsor’s involvement remains aligned with the study objectives.

### Background and rationale {6a}

Cerebral palsy (CP) is a neurodevelopmental disorder that represents the most common cause of disability in childhood [1,2]. Spastic diplegia is the most prevalent form of CP, affecting over 50% of patients. It often results in a characteristic walking pattern known as crouch gait, which primarily arises from weakness predominantly in the extensor muscles and spasticity mainly affecting the flexors. This gait pattern is characterized by excessive knee flexion during the stance phase, often accompanied by insufficient hip extension and excessive ankle dorsiflexion, and sometimes accompanied by increased hip flexion and adduction, internal rotation, and various ankle alignment (plantarflexed, neutral, or dorsiflexed), which together can progressively impair walking [3,4]. Although many children with spastic diplegia achieve independent walking, their gait often deviates significantly from age-typical patterns [5], with or without the use of assistive devices. While CP itself is non- progressive, approximately 50% of individuals who walk during adolescence lose this ability by early to mid- adulthood due to progressive musculoskeletal complications, including the worsening of crouch gait. Primary impairments such as weakness, spasticity, and impaired motor control are present from early development, whereas secondary weakness and reduced endurance may arise from decreased physical activity, further contributing to functional decline [6]. Together, these factors affect daily function and substantially reduce quality of life.

Multiple factors contribute to crouch gait, including muscle spasticity, contractures, poor motor control, dystonia, muscle imbalance, and weakness, which can affect the ankle, knee, or hip joints at different levels. The relative contribution of each factor varies among individual. In cases where muscle contracture is the primary contributor to crouch gait, orthopaedic surgery may be required to lengthen the muscle-tendon unit. However, careful and personalized consideration is always necessary to avoid excessive lengthening, which could weaken muscles and potentially exacerbate crouch gait [7]. Beyond contracture-related issues, extensor muscle weakness is nearly universal in children with CP and, although rarely an isolated cause, it plays a key role in the progression of crouch gait, particularly as body mass increases with growth while muscle strength fails to keep pace [8]. Weakness in key extensor muscle groups, including the hip extensors, knee extensors and ankle plantar flexors, compromises the ability to maintain upright posture and functional mobility [9]. This is often associated with lever arm dysfunction of the knee extensor mechanism during loading [10]. Traditional ankle-foot orthoses (AFOs), commonly prescribed to support gait, provide passive stabilization but may contribute to further leg muscle weakening over time due to disuse [11]. Consequently, there is an urgent need for interventions that not only assist walking but also promote active engagement and muscle strengthening to support long-term mobility and functional independence.

Over the past several decades, electromechanical devices have been used for gait training in paediatric patients with neurological disorders. Body-weight-supported treadmill (BWST) systems have shown varying degrees of effectiveness in gait rehabilitation for children with CP [12], while advanced robotic exoskeletons with BWST systems (e.g., LOKOMAT by Hocoma Inc.) provide a safe and controlled environment for gait training [13]. In recent years, several studies have investigated the effectiveness of robotic exoskeletons in gait rehabilitation for children with CP. Pilot trials in hospital settings have reported improvements in gait quality both with the use of wearable exoskeletons [14] and weight supported devices [15]. A case study reported the applicability of a robotic-assisted walker in helping a child with quadriplegic CP to walk overground [16]. Experimental studies have shown that motorized exoskeletons can immediately improve knee extension, walking speed and stride length, providing controlled assistance during the stance and swing phases [17,18]. Other works suggest that exoskeleton training may promote neuromuscular adaptations and long-term functional improvements, although further studies are needed to confirm these effects [19,20]. Finally, a recent review emphasized that robotic exoskeletons have the potential to improve mobility in children with CP, increasing community participation and quality of life, and it highlighted the need for larger controlled intervention studies [21].

These findings have sparked growing interest in long-term studies on robotic exoskeletons for gait rehabilitation in children with CP, including their potential application in home settings. Preliminary research in adults suggests that home-based exoskeleton training is feasible and may lead to functional improvements, although challenges regarding device usability, long-term adherence, and real-world effectiveness remain unsolved [22].

In this context, where there is an increasing demand for lightweight and accessible exoskeletons for home rehabilitation, Bionic Power Inc. developed Agilik™, a wearable exoskeleton designed to provide dynamic knee assistance and resistance during gait, depending on each child’s motor impairments and therapeutic goals. The setting mode is based on baseline gait analysis and clinical assessment, with assistance applied when knee extensor weakness predominates and resistance used to strengthen the extensors and improve motor control. The device, CE marked and approved by the FDA, has already demonstrated its effectiveness in a small sample of patients [23]. Recent studies, including those by Devine et al. [24], have explored the feasibility of using Agilik™ in home-based rehabilitation, but further research is needed to establish its long-term impact in real-world settings.

The study described in this protocol aims to address this gap by evaluating the long-term effects of the Agilik™ device in a home setting. Unlike previous investigations, which have often focused on short-term hospital-based interventions, this trial assesses functional and clinical outcomes after training in a real-world environment. Similar to Devine et al. [24], we evaluate gait training with a robotic exoskeleton outside of the clinical setting, following an initial in-clinic accommodation period. However, unlike their study, the present trial also includes a comprehensive evaluation of usability, acceptability, and user experience, incorporating quantitative and qualitative feedback from both patients and caregivers to provide essential insights into the real-world applicability of the technology.

### Objectives {7}

The primary objective of this study is to evaluate the efficacy of Agilik in improving anti-gravity knee extension and overall endurance in a cohort of individuals with CP and crouch gait, in a home-based setting compared to usual care.

The secondary objective is to assess differences between the Intervention Group (receiving Agilik device training) and the Control Group (continuing with usual care and daily activities) in terms of joint range of motion (ROM), muscle length, balance, spasticity, postural and motor abilities, gait speed and pattern, and self-perceived performance.

The third objective is to evaluate the usability and acceptability of the exoskeleton in the Intervention Group, along with user experience and engagement from the perspectives of both patients and caregivers.

Lastly, as an exploratory outcome, gait behavior will be remotely monitored in a selected group of children using sensorized shoes (NUSHU, MAGNES AG, Hardturmstrasse 253, 8005 Zurich) to track spatiotemporal parameters in an ecological setting.

### Trial design {8}

The clinical investigation is designed as a Randomized Controlled Trial (RCT) with a parallel-group structure. Participants will be randomly assigned to either the Intervention Group, which will receive the Agilik device for home-based gait training, or the Control Group, which will not receive the device. Both groups will continue to follow their usual care and conventional rehabilitation treatments if previously prescribed. In Italy, conventional treatment may vary across regions and age groups ranging from minimal to very intensive rehabilitation. Despite the heterogeneity, usual care is the most suitable and clinically valid benchmark, since it corresponds to the real-world management of crouch gait in children with CP. The allocation ratio will be 1:1, ensuring equal numbers of participants in both groups.

Additionally, the trial is confirmatory and not first-in-human study, as there is existing preliminary evidence supporting the device’s effectiveness. It will be conducted prospectively, observing outcomes over time from baseline (T0) through both the intervention and follow-up phases. The multicentre nature of the study, conducted across seven clinical centres, will help ensure a broader and more representative sample of participants, thereby increasing the generalizability of the findings.

## Methods: Participants, interventions and outcomes

### Study setting {9}

The study will be conducted in clinical and home settings across multiple specialized centres. Data will be collected in Italy from seven rehabilitation centres, affiliated with four specialized institutions dedicated to the treatment of children with cerebral palsy and other neuromotor and neurodevelopmental conditions. These institutions include IRCCS E. Medea, IRCCS Fondazione Stella Maris, Fondazione Don Carlo Gnocchi Onlus (Milano, Rome – two centres – and Salerno), and IRCCS Fondazione Mondino. Reference to the list of study sites can be found in the table of administrative information. Participant recruitment began on 29/10/2024 and is expected to be completed by 31/07/2026.

### Eligibility criteria {10}

We plan to enrol 40 children with CP exhibiting crouch gait, as determined by sample size calculation.

#### Inclusion criteria:

- Children diagnosed with CP, specifically with spastic diplegia or a bilateral spastic form characterized by greater involvement of the lower limbs compared to the upper limbs, and exhibiting crouch gait.
- Age between 5 and 17 years.
- Body weight between 20 and 125 kg.
- Knee flexion contracture of less than 10° in the supine position. Hamstring contracture assessed by the straight leg raising test must not limit participation in the study.
- No tibio-tarsal arthrodesis, and at least 5° of passive ankle dorsiflexion.
- Ability to walk at least 3 meters without stopping, with or without a walking aid.
- Ability to understand and follow simple directions, as confirmed by parent report and physician observation.
- Gross Motor Function Classification System (GMFCS) level I, II, or III.
- Modified Ashworth Scale (MAS) score ≤ 2 for each segment of the lower limb.
- Provision of signed and dated informed consent form by legal guardian.
- Willingness to comply with all study procedures and availability for the study duration, or demonstrated ability to do so as determined by parent report and physician observation during history and physical examination.

#### Exclusion criteria:

- Severe neurological, musculoskeletal, or cardiorespiratory conditions that prevent walking.
- History of uncontrolled seizures within the past year.
- Severe spasticity.
- GMFCS level IV and V.
- Hip or knee flexion contracture exceeding 20°.

### Who will take informed consent? {26a}

Each participant will be officially enrolled in the study upon providing informed consent by their legal guardian. Recruitment will take place at the following clinical centres: IRCCS E. Medea (Bosisio Parini), IRCCS Fondazione Stella Maris (Calambrone), IRCCS Fondazione Mondino (Pavia), Fondazione Don Carlo Gnocchi Onlus – Centro Santa Maria della Provvidenza (Rome), Fondazione Don Carlo Gnocchi Onlus – Centro Santa Maria della Pace (Rome), Fondazione Don Carlo Gnocchi – Centro S. Maria al Mare (Salerno), and Fondazione Don Carlo Gnocchi – IRCCS S. Maria Nascente (Milano). All participants will receive a verbal explanation suited to their level of comprehension of the purposes, procedures and potential risks of the study and of their rights as research participants. Minors will also receive an information sheet explaining the study procedures, one version for the age group 6–11 and another for the age group 12–17. Participants’ guardians will have the opportunity to carefully review the written consent form and ask questions regarding this study prior to signing.

### Additional consent provisions for collection and use of participant data and biological specimens {26b}

n/a. In this study, additional consent provisions for the collection and use of participant data and biological specimens in ancillary studies are not applicable.

### Ethics approval and consent to participate {24}

The study protocol, including informed consent forms, privacy notices, and all relevant documentation pertaining to the protection of participants’ rights and data, was submitted to the Comitato Etico Territoriale Lombardia 2 for review and approval. The committee’s approval was secured prior to the commencement of any participant recruitment or data collection activities, in full compliance with national regulations and ethical standards (n. R1927/24 – L2-131, 19/06/24). Any subsequent amendments to the study protocol will be promptly submitted to the same ethics committee for further review and approval if required.

Written, informed consent to participate will be obtained from all participants.

## Interventions

### Explanation for the choice of comparators {6b}

The choice of comparators in this study is based on the need to evaluate the clinical effectiveness of the Agilik device after a two-month home-based treatment compared to standard care for children with CP exhibiting crouch gait.

The chosen comparator is the usual standard therapy, which in Italy is known to be highly variable depending on the territory, ranging from minimal to very intensive rehabilitation. Despite this variability, usual care is the most appropriate and clinically relevant comparator, as it reflects the real-world therapeutic context in which children with crouch gait due to CP are typically treated. All rehabilitation activities, including type, frequency, and amount of standard therapy, will be systematically recorded for every participant, ensuring that this information can be accounted for in the statistical models as covariate. This will allow to adjust for differences in overall rehabilitation exposure across participants and to isolate the specific effect of the Agilik intervention, thereby ensuring that the estimated treatment effect is not confounded by variability in routine care.

### Intervention description {11a}

This study aims to demonstrate the clinical benefits of the Agilik exoskeleton in children with CP and knee extension deficiency. Participants will be selected based on inclusion/exclusion criteria and their medical/physical history. Once eligibility is confirmed, demographic data (such as age, gender, height, weight, diagnosis, and GMFCS level) will be collected. Eligible children will be randomly assigned to one of two groups: the Intervention Group or the Control Group, with randomization stratified by GMFCS level and gender to help balance population heterogeneity. For ethical reasons, withholding standard therapy would not be acceptable in this population. Therefore, participants may continue their usual standard therapy, if already prescribed. All participants will begin the study after both parents or legal guardians have signed the informed consent form.

The Agilik is a powered orthosis system designed to assist or resist knee motion independently in each phase of gait. It is typically used as a bilateral device, with one orthosis for each leg. Each unit applies up to 12 Nm of torque at the knee joint in either flexion or extension. The system comprises patient-specific custom-molded Knee-Ankle-Foot Orthoses (KAFOs) with integrated electro-mechanical actuators, a battery, a carry pack, cabling, and dedicated software (Agilik App). A foot pressure sensor embedded in the footbed of the KAFO and connected to the actuator, together with measurements of knee angular velocity, allows real-time detection of the gait phase. This enables the motor controller to deliver phase-specific torque assistance or resistance. According to the user manual, assistance is provided during initial and mid stance as well as late swing, while resistance is applied during initial swing. Additionally, depending on the individual gait pattern, assistance or resistance may also be provided during late stance. Personalization is achieved through the Agilik App, which allows therapists and biomedical engineers to fine-tune torque parameters according to the child’s gait pattern, knee strength, comfort level, and therapeutic goals. The torque is progressively calibrated during clinical sessions to ensure that patients train effectively without overexertion.

The Agilik is classified as a Class I medical device for both clinical and home use and is rated for non-continuous operation under IEC 60601−1.

The first visit for participants in both the Intervention and Control Groups will include a physical examination and baseline (T0) assessments, which include:

- 6MWT to evaluate gait endurance
- KEMS to measure knee active extension during overground gait analysis
- Gross Motor Function Measure-88 (GMFM-88) for gross-motor assessment
- 10-Meter Walk Test (10mWT) for functional gait assessment
- Modified Ashworth Scale (MAS) to evaluate spasticity in the lower limbs
- Joint ROM and muscle length assessments, conducted manually by a therapist
- 3D gait analysis with Electromyography (EMG) overground
- Centre of Pressure (COP) detection to quantify balance
- COPM (Canadian Occupational Performance Measure) for self-perceived performance.

Following the baseline assessments, the Control Group will continue with daily activities potentially including standard care according to their prescription. Participants of both groups will be asked to wear the NUSHU sensorized shoes (MAGNES AG, Hardturmstrasse 253, 8005 Zurich) during a daily 2-minute walking test when feasible.

The Intervention Group will undergo a structured program with the Agilik device, consisting of the following phases:

- **Phase 1: Exoskeleton Preparation**. During up to three visits, the custom casting for the KAFO will be completed for participants.
- **Phase 2: System Setup, Calibration, and Training**. During this phase, which lasts 4–5 weeks with a minimum of two sessions per week, participants will practice walking with the device under clinical supervision. During this period, torque assistance and resistance are tailored to each participant, based on repeated gait evaluations and real-time feedback from the clinical and engineering team. Families will also receive training to use the system independently at home, ensuring they are comfortable and confident with device operation and management.
- **Phase 3: Home-Based Intervention**. Participants will use the Agilik™ at home for two months, 30 minutes per day, five days per week. The training dosage is defined in collaboration with the clinical team, based on standard in-clinic intensive rehabilitation protocols in Italian centres, which typically include five 45-minute sessions per week. Considering that the Agilik™ sessions are conducted at home under caregiver supervision rather than with a therapist, the duration is reduced to 30 minutes per session to ensure an appropriate yet sustainable workload. Participants are instructed to perform the training sessions either inside the home or outdoors in a safe environment, according to their preference. The use of the device is not restricted to indoor spaces, although caregivers are advised to avoid use during rainy weather or any conditions that could expose the device to moisture. Walking on both flat and sloped surfaces (uphill or downhill) is permitted, with no specific environmental restrictions beyond safety considerations. Participants must always be accompanied by a caregiver during device use. While they are encouraged to complete at least five 30-minute sessions per week, they are allowed to train more frequently or for longer durations if they wish. The training protocol remains fixed throughout the two-month home intervention period. Only minor adjustments to the app parameters are made, and a new prescription is provided if gait deviations are identified during monitoring sessions.

In case of technical problems with the device, corrective actions will be implemented. During the in-clinic training phase, families are instructed on basic troubleshooting procedures to manage minor issues independently. Biomedical engineers from the research team will provide support to resolve minor problems, while the Italian distributor (Orthoservice Ro + Ten) or the manufacturer (Bionic Power) will be involved if technical issues persist. All device malfunctions, along with any interruptions in the training program and their duration, will be documented to account for their potential impact during data analysis. If the training is interrupted for an extended period, the participant will be considered a dropout.

At T1 (about 3 months after T0), both groups will undergo outcome assessments, and follow-up assessments will take place one month later (T2). Follow-up assessments will mirror the T0 evaluations, while T1 outcome assessments for the Intervention Group will additionally include the System Usability Scale (SUS) and the Technology Acceptance Model (TAM-3) to evaluate the usability and acceptability of Agilik. At T1, semi-structured interviews will be conducted with each family in the Intervention Group. These qualitative insights will be integrated with information gathered from the daily diaries to explore user experiences, perceived ease of use, challenges encountered, and to provide a richer understanding of patient engagement throughout the home-based intervention.

### Criteria for discontinuing or modifying allocated interventions {11b}

Participants (or their legal guardians) have the right to withdraw from the study at any time due to personal reasons or discomfort. In all cases, decisions regarding discontinuation of interventions will be made by the study team in consultation with the participant and based on the study protocol and ethical guidelines.

### Strategies to improve adherence to interventions {11c}

To enhance participant adherence to the study protocol, several strategies will be implemented. First, detailed training will be provided to both participants and their caregivers before the trial begins. This comprehensive training will cover the proper use and maintenance of the Agilik device, emphasizing the importance of adherence to the study procedures.

Regular support will be integral to the study, involving phone calls and/or virtual check-ins. These interactions will offer ongoing assistance, address any questions or concerns, and reinforce the significance of adhering to the intervention protocol.

Clear instructions and reminders will be provided through both written and verbal communication. Participants will receive detailed guidelines on the intervention procedures, including the use of the Agilik device and any required assessments. Additionally, reminders regarding upcoming visits, device usage, and other protocol-related activities will be communicated via email, text messages, or phone calls.

For younger participants, caregivers will play a crucial role in the training and adherence process. Their involvement and support will be essential to ensure that participants consistently follow the protocol throughout the trial.

To effectively monitor adherence throughout the trial, participants and their families will keep a diary to document engagement with the device. They will detail their experiences with the device and any challenges they may have faced. This information will help ensure adherence and allow the study team to identify and address any issues promptly.

### Relevant concomitant care permitted or prohibited during the trial {11d}

Permitted concomitant care and interventions include standard medical care, where participants may continue with their routine medical visits and necessary treatments that do not interfere with the study protocol. The use of orthotic devices or walking aids, excluding those specifically related to the Agilik device, is permitted as long as they do not interfere with study assessments or the functionality of the device. Surgical and/or pharmacological interventions targeting knee extension or spasticity that could affect study outcomes are not permitted during the trial unless necessary for the participant’s safety or medical condition.

### Provisions for post-trial care {30}

In the event that a participant suffers harm due to trial-related procedures, appropriate medical care will be offered. Participants will also be provided with information on how to have further care, if needed, after the trial concludes. The sponsor has taken out insurance valid for all participants in accordance with current regulations.

### Outcomes {12}

To demonstrate the effectiveness of Agilik during to the home-based training, we will evaluate the following primary endpoints at baseline (T0), post-intervention (T1), and follow-up (T2):

- Distance walked during the 6MWT [25]: this standardized test measures the maximum distance a subject can walk in six minutes to evaluate gait endurance. According to Guinet *et al.* [26], the test-retest reliability of the 6MWT is excellent in individuals with cerebral palsy (intra-class correlation coefficient: 0.87–0.98).
- Knee extension in mid-stance (KEMS) during gait analysis: this parameter assesses the effectiveness of the device in improving knee extension during walking, focusing on joint alignment and function in the gait cycle. KEMS is calculated as the average of the maximum knee extension achieved during the mid-stance phase across multiple trials for both the right and left lower limbs. The mid-stance phase occurs when the support limb transitions from shock absorption to stability. It is defined as the period from the toe-off of the contralateral leg to the moment when the heel of the leading leg lifts off the ground.

Secondary endpoints will be assessed at baseline (T0), post-intervention (T1), and follow-up (T2) and are reported in the following section:

- Joint ROM and muscle lengths of the lower limbs [27]. These measures evaluate potential changes in joint mobility and muscle extensibility using manual goniometry and physical assessment techniques. According to a pilot study [28], goniometric measurements in children with CP demonstrated excellent intra-examiner reliability (>0.80), while inter-examiner reliability was lower (0.375 and 0.475).
- Spasticity of the lower limb muscles will be evaluated using the MAS [29]. The MAS is used to grade the resistance of muscles to passive stretch. According to Ansari *et al*. [30], it shows moderate inter-rater (0.514) and intra-rater (0.590) reliability.
- Postural and gross motor abilities assessed with the GMFM-88 [31]. This validated scale measures motor skills, such as sitting, standing, and walking, to track functional changes over time. According to Nordmark et al. [32], the GMFM is a reliable instrument for assessing motor function and evaluating treatment outcomes in individuals with cerebral palsy (inter-rater reliability: 0.77; intra-rater reliability: 0.68).
- Functional gait measured by the 10mWT [33]. Walking speed is recorded over a distance of 10 meters, providing insights into functional ambulation capacity. A study found that in youths with neurological gait disorders [34], the intra-class correlation coefficient of the 10MWT at the preferred velocity ranged from 0.81 to 0.95, indicating high reliability.
- Gait kinematics and kinetics will be evaluated in a 3D gait analysis laboratory with EMG data acquisition. This method uses 3D motion capture and a 4-channels surface EMG system to assess gait patterns and muscle activation, providing a detailed look at the biomechanics of walking. The laboratory features an optoelectronic motion capture system with infrared cameras. If available in the gait laboratory of the rehabilitation centre, two force plates embedded in the floor will record ground reaction forces. Surface EMG will be acquired bilaterally from the rectus femoris and semimembranosus muscles. To evaluate changes in muscle function, EMG-derived outcome measures will include activation timing parameters and co-contraction indices, among the standard metrics reported in the literature for assessing muscle activation patterns in children with CP [35–37]. Data collection and analysis will follow the Davis protocol [38] or the LAMB protocol [39,40], depending on the standard procedures adopted by each rehabilitation centre. After the application of the markers directly on the patient’s skin, each subject will be asked to walk barefoot along a 10-metre walkway. Studies on children with CP have demonstrated good reliability of 3D gait analysis [41], with a mean standard error of measurement of 3.0° across all analysed models and joint angles.
- Balance quantified using COP detection [42]. A force platform will be used to assess postural control by tracking COP displacement during static standing. Data will be collected under four conditions: eyes open with feet apart, eyes open with feet together, eyes closed with feet apart, and eyes closed with feet together. Each condition will be measured three times for 60 seconds while the participant is standing.
- Self-perceived performance assessed with the COPM [43]. This tool allows participants (and their caregivers) to rate the importance and performance of various activities in daily life, providing insight into their perception of functional abilities. The COPM has established reliability, validity, and responsiveness to change. It has been translated into over 35 languages and it is widely used across various health conditions [44].

Additionally, at T1, the following assessments will be conducted exclusively for the Intervention Group:

- Usability and acceptability evaluated using the SUS and TAM-3:
  - SUS [45] evaluates the usability of the system by using 10 questions, scored on a 5-point Likert scale. The final score ranges from 0-100, indicating the overall perceived usability of the device. The SUS has demonstrated high reliability [46], with a Cronbach’s alpha of ≥0.90, ensuring consistent and reproducible usability assessments across different user groups.
  - TAM-3 assesses the acceptability of the device through 50 questions grouped into 14 items (e.g., “perceived usefulness,” “perceived ease of use,” “self-efficacy,” “anxiety,” etc.). Responses are evaluated on a 7-point Likert scale, with the overall score calculated as the mean of the items. Venkatesh *et al.* [47] have confirmed the reliability and validity of TAM-3 in measuring behavioural intention and actual technology use.
- Behavioural and cognitive engagement will be explored through semi-structured interviews with the patient’s caregiver, based on an ad hoc structure specifically developed for this protocol. The interviews aim to understand the child’s interaction with the Agilik device, adherence to the training program, and caregiver attitudes and perspectives on both current use and potential future applications.

Parents and caregivers in the Intervention Group will also be provided with a diary to record their observations and feelings about the training period, facilitating a more comprehensive monitoring of the treatment (e.g., use scenarios, and/or challenges encountered) and the patient’s emotional engagement.

Finally, as an exploratory outcome, we will perform remote monitoring of gait function using sensorized shoes (NUSHU, MAGNES AG, Hardturmstrasse 253, 8005 Zurich). Participants in both groups will be asked to wear these shoes every day during 2-minute indoor walking, if feasible, to collect data on gait spatiotemporal parameters during daily life.

Data for the study will be gathered and managed with REDCap electronic data capture tools. REDCap (Research Electronic Data Capture) is a secure, web-based software platform designed to facilitate data collection for research projects. It offers 1) a user-friendly interface for validated data entry; 2) audit trails to track data modifications and export actions; 3) automated export features for easy data transfer to common statistical software; and 4) integration capabilities to enable interoperability with external data sources [48,49].

### Participant timeline {13}

The trial will consist of several phases including enrolment, baseline assessments, an intervention period, outcome assessments and follow-up assessments. Below is the timeline and description of key activities at each stage.

1. **Enrolment and Baseline (T0)**

The enrolment phase involves screening potential participants to ensure they meet the inclusion and exclusion criteria. Once eligibility is confirmed, demographic data (such as age, sex, height, weight, diagnosis, and GMFCS level) will be collected. After both legal guardians have provided signed informed consent, the enrolled participant will be randomly assigned into one of two groups: the Intervention Group or the Control Group. This randomization will be stratified based on GMFCS level and gender, ensuring balance in these factors across the study. Baseline assessments will then be conducted to collect initial clinical data (i.e., 6MWT, KEMS, joint ROM, muscle lengths evaluation, MAS, GMFM-88, 10mWT, 3D Gait Analysis with EMG, COP detection, COPM). These assessments will provide the data for subsequent comparisons throughout the study.

2. **Intervention**

After Baseline assessments, the Control Group will continue with standard therapy and daily life activities, while the Intervention Group will start the intervention, which consists of exoskeleton preparation, Agilik training in a clinical setting (4–5 weeks) and home-based training (2 months) as described in section “Intervention description”.

Both Intervention and Control Group participants will perform a two-minute walking test every day wearing sensorized shoes, when feasible, to monitor gait data.

3. **Outcome Assessments (T1)**

To evaluate the long-term effects of the Agilik device, assessments will be conducted at T1, about 3 months from baseline. At this stage, both groups will undergo the same assessments as at T0. For the Intervention Group, additional assessments will be made on the usability and acceptability of Agilik through the SUS and the TAM-3 questionnaires. Semi-structured interviews will be conducted to gather qualitative feedback from patients’ caregivers regarding their experience with the device.

4. **Follow-up Assessments (T2)**

One month after T1, a final assessment will be conducted mirroring the baseline assessment (T0), to track any sustained improvements, regressions, or maintenance of the status one month after discontinuing Agilik use.

**Fig 1:**
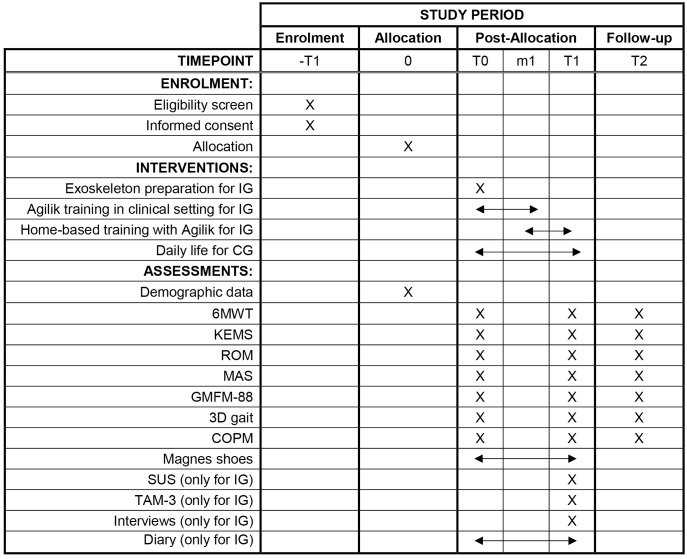
Flow of activities and assessments in the Agilik@home protocol. IG: Intervention Group; CG: Control Group. T0: baseline assessments; m1: 4–5 weeks after baseline; T1: approximately 3 months after baseline; T2: one month after T1.

The timeline in Fig 1 illustrates the flow of activities and assessments to ensure comprehensive monitoring of each participant’s progress during the trial.

### Sample size {14}

We selected standardized effect sizes based on the published literature and clinical relevance. For the 6MWT we assumed a moderate standardized effect size of 0.4, consistent with effect sizes reported in similar paediatric rehabilitation studies [13,50], and we used a clinically meaningful threshold of 25 m as the minimum important difference. For the KEMS we assumed a larger standardized effect (1.0) supported by previous instrumented studies and pilot data on knee-extension–targeted interventions [51]. The trial is powered to detect a significant difference in at least one of the two co-primary endpoints using the approach described by Sozu *et al.* [52]; a conservative correlation of 0.5 between the two endpoints was assumed. With α = 0.025 (two-sided) and power = 0.80, these parameters yield a required sample of 18 participants per group (36 total). To account for potential dropout (estimated at 10%), the total sample size is conservatory increased to 40 participants. Sample size calculations were performed in R.

### Recruitment {15}

The study will be conducted across multiple specialized centres to increase the pool of potential participants. Subjects with CP who potentially meet the inclusion criteria will be identified by clinicians at the participating clinical centres. Physicians will be informed about the study and encouraged to refer suitable candidates.

Recruitment progress will be regularly monitored, and adjustments will be made to the recruitment strategy if necessary, based on enrolment rates at each site.

Finally, patient retention measures will be implemented through regular communication, follow-up calls, and ongoing support provided to participants and their families. This approach aims to ensure that once enrolled, participants remain committed to completing the study.

## Assignment of interventions: Allocation

### Sequence generation {16a}

40 participants will be enrolled, 20 in the Intervention Group and 20 in the Control Group. To balance the groups based on critical variables that could influence the study outcomes, participants will be stratified according to two key factors:

1. **GMFCS Level**: participants will be categorized based on their GMFCS level to ensure an even distribution of motor impairment between groups.
2. **Gender**: stratification by gender will help avoid any potential bias caused by gender differences in the response to the intervention

Therefore, a 2x3 table (gender x GMFCS) was generated, assigning 4 participants per group for each combination. Then, each sequence was permuted to achieve a random assignment of participants while maintaining the pre-determined group sizes.

### Concealment mechanism {16b}

As described in the “Sequence generation” section, the allocation sequence was predetermined centrally. To maintain complete concealment, the study team will not have access to the allocation information prior to the intervention assignment. This will ensure that neither participants nor the research team could influence or anticipate the assignment to the Intervention or Control Group, minimizing the risk of bias during the study. The concealment will be maintained until the intervention is assigned to each participant.

### Implementation {16c}

The allocation sequence is centralized across all participating centres and generated as described in section “Sequence generation”. The randomization process will be conducted immediately after the informed consent is signed. Study investigators responsible for enrolment at each clinical site will not have access to the allocation sequence at any stage, ensuring complete allocation concealment and minimizing the risk of bias.

## Assignment of interventions: Blinding

### Who will be blinded {17a}

Due to the nature of the intervention (use of the Agilik orthosis), it is not feasible to blind the participants, the assessors and the care providers delivering the intervention. However, all efforts will be made to ensure that care providers deliver the intervention consistently across the Intervention Group and without bias.

### Procedure for unblinding if needed {17b}

n/a. In this trial, unblinding procedures are not applicable because the nature of the intervention (use of the Agilik device) makes it impossible to blind participants and caregivers. Additionally, for organizational reasons, care providers will also be aware of the group assignments.

## Data collection and management

### Plans for assessment and collection of outcomes {18a}

Demographic and medical history data, as well as specific motor parameters relevant to the study (e.g., degree of knee extension deficiency, GMFCS level), will be collected during the initial screening. Baseline functional measures will be conducted to capture participants’ physical status before the intervention, and primary and secondary outcomes will be assessed at baseline (T0), post-intervention (T1), and follow-up (T2).

Trained staff, with an average of 10 years of experience, will perform assessments using standardized protocols, calibrated instruments and validated tools to ensure reliability. Assessors involved in data collection will receive comprehensive training on the proper use of each tool to maintain consistency in data recording and minimize variability between raters.

To ensure consistency across all participating centres, outcome measures and data collection procedures will be standardized and shared among the centres. Each centre will follow the same protocols for measuring outcomes and documenting results.

Data will be recorded in real time and uploaded to the REDCap electronic data capture system, which incorporates validation rules to identify data entry errors and highlight missing values. This approach will support continuous data quality monitoring and help maintain the integrity of the collected data throughout the trial.

### Plans to promote participant retention and complete follow-up {18b}

Regular communication with participants and their caregivers will be maintained to emphasize the importance of follow-up visits and assessments. To minimize missed appointments, reminders via email, phone calls, or text messages will be sent prior to each assessment or visit. Additionally, appointment times will be flexible to accommodate the daily lives of participants, thereby facilitating their attendance.

Efforts will be made to foster positive relationships with participants by providing ongoing support throughout the intervention period. Addressing any concerns and recognizing their contributions to the research will further encourage their engagement. Importantly, the strategies employed to retain Control Group participants will mirror those used for the Intervention Group, ensuring equal emphasis on communication, reminders, and support for completing follow-up assessments.

If a participant discontinues the intervention, the reasons for withdrawal will be investigated. Any adverse events will be recorded, and all available data collected up to the point of discontinuation will be retained. Participants who discontinue will continue to be monitored for any adverse events related to the intervention, adhering to ethical and safety requirements.

To address missing data, appropriate statistical methods, such as multiple imputation under the assumption of missing at random, will be utilized, particularly for primary outcomes, ensuring that the analysis remains robust despite any participant dropouts.

### Data management {19}

Data entry will primarily be conducted using REDCap, a secure electronic data capture system that minimizes human error and facilitates efficient data management. By utilizing REDCap, we aim to streamline the data collection process while upholding the highest standards of data security and confidentiality.

The coding of participant data will be performed at each participating centre using secure and standardized procedures to ensure confidentiality. A unique identification code will be generated for each participant based on personal information (e.g., tax code, name, surname, gender, date, and place of birth), and personal identifiers will be stored separately from coded data. Access to sensitive information will be restricted to authorized personnel only, following secure login protocols.

Data security will be a top priority. Access to digital platforms such as REDCap will be restricted to authorized team members, ensuring that sensitive information is handled responsibly. Data storage will occur in secure servers located within a controlled environment, with regular backups to prevent data loss. Paper records will be stored in locked cabinets accessible only to designated team members.

Training for data collectors will be a key component of the study preparation. A dedicated training session will be conducted during the project’s kick-off meeting to ensure all personnel are well-versed in data management procedures and fully understand the importance of maintaining data integrity.

### Confidentiality {27}

Personal information will be collected through informed consent forms, demographic questionnaires, and clinical assessments. All forms were designed to collect only the necessary information required for the trial, minimizing the risk of unnecessary exposure of sensitive data. Upon collection, personal information will be pseudonymized and a unique identification will be assigned to each participant to produce a unique code used throughout the trial for all research documentation and data analyses.

Personal identifiers, such as names and contact details, will be securely stored separately from the coded data. This ensures that personal identifiers are not linked to the coded data in any manner that could compromise confidentiality.

Access to personal information will be further restricted to authorized personnel only, including research team members directly involved in the study. Any sharing of information, whether within the research team or with external parties, will be conducted in accordance with strict data sharing protocols and confidentiality agreements, ensuring that data is shared solely for legitimate research purposes and with appropriate safeguards in place.

All data will be securely stored in REDCap, a password-protected electronic system that complies with data protection regulations. Physical documents containing data will be kept in locked cabinets accessible only to authorized personnel.

Participants will be informed of their rights regarding their personal information, including the right to access, modify, or withdraw their data at any time. Clear communication will be maintained throughout the trial to ensure participants understand how their information is being used, shared, and protected.

### Plans for collection, laboratory evaluation and storage of biological specimens for genetic or molecular analysis in this trial/future use {33}

n/a. The collection, laboratory evaluation, and storage of biological specimens for genetic or molecular analysis are not applicable to this trial, since no biological specimens will be collected as part of the trial, and there are no plans for genetic or molecular analysis either during the trial or for future ancillary studies.

## Statistical methods

### Statistical methods for primary and secondary outcomes {20a}

Statistical analyses for both primary and secondary outcomes will be conducted using SPSS statistical software. The primary outcomes (6MWT and KEMS) will be analysed to evaluate changes over time and differences between the Intervention and Control Groups. Demographic data and dropout rates will be summarized using descriptive statistics. Data collected during outcome assessments and follow-up will be compared with baseline (T0) measurements for intra-group analysis. Additionally, comparisons will be made between the Intervention and Control Groups for inter-group analysis.

Qualitative data (collected through the semi-structured interview and the daily diary) will be analysed according to the inductive thematic analysis and using the Atlas.ti software.

The normality of the dataset will be assessed using appropriate statistical tests, such as the Shapiro-Wilk test. Based on these results, parametric or non-parametric methods will be applied. If the data follow a normal distribution, a mixed-design ANOVA will be employed to assess changes over time within each group and between groups, and to explore potential interaction effects. For non-normally distributed data, non-parametric alternatives will be used: the Friedman test for within-group comparisons across time points, and the Mann–Whitney U test (or Kruskal–Wallis test, if needed) for between-group comparisons at each time point. Overall therapy dosage will be included as a covariate in the data analysis to ensure that the study evaluates the efficacy of Agilik under real-life clinical conditions and in comparison with the care these children routinely receive.

For detailed information regarding the calculation of the sample size, refer to the *Sample Size* section.

### Interim analyses {21b}

No interim analyses have been planned for this trial. As such, there are no predefined stopping guidelines. The trial will proceed according to the established protocol until its completion, unless unforeseen circumstances necessitate a review. The decision to terminate the trial, if necessary, will be made by the principal investigator, in consultation with the relevant ethics committee and other stakeholders, based on the overall safety and integrity of the study.

### Methods for additional analyses (e.g., subgroup analyses) {20b}

In addition to the analyses on primary and secondary, additional analyses will be conducted to explore specific subgroups and adjust for potential confounding factors. Subgroup analyses will be performed to assess whether the intervention has different effects on participants based on baseline characteristics, such as gender, age, GMFCS level, or walking ability with or without assistive devices.

### Methods in analysis to handle protocol non-adherence and any statistical methods to handle missing data {20c}

The primary analysis population will follow the “intention-to-treat” (ITT) principle, meaning that all participants will be analysed according to the group to which they were initially randomized, regardless of their adherence to the intervention protocol. This ensures that the analysis remains unbiased and maintains the benefits of randomization, allowing for a more accurate representation of the intervention effectiveness in a real-world scenario.

To handle missing data, appropriate statistical methods will be applied to minimize potential bias and preserve the robustness of the analysis. Missing data may result from participants discontinuing the intervention, not completing follow-up assessments, or having incomplete outcome measurements (e.g., due to technical issues with measurement devices). The approach to missing data will depend on both the underlying mechanism (missing at random, missing completely at random, or missing not at random) and the amount of missingness. Based on these factors, suitable techniques, such as multiple imputation, exclusion of unreliable observations, or the use of linear mixed models, will be employed to ensure that the analyses remain valid and interpretable.

### Plans to give access to the full protocol, participant level-data and statistical code {31c}

The full study protocol will be published ensuring that the methodology is fully accessible to the research community. Regarding the participant-level dataset, it will be uploaded in online repository such as Zenodo.org and access will be provided following the completion of the trial and publication of the results, subject to ethical and privacy considerations. The dataset will be anonymized to protect participant confidentiality. Details regarding the procedures for requesting access to the dataset will be included in the final publication.

## Oversight and monitoring

### Composition of the coordinating centre and trial steering committee {5d}

IRCCS E. Medea acts as the coordinating centre for the trial. The coordinating centre is responsible for the overall management and oversight of the trial. It acts as the central hub for communication and logistical support. The coordination centre is in charge of overseeing the implementation and conduct of the trial in accordance with the protocol, and ensuring that the trial adheres to regulatory requirements and ethical standards.

Each participating site will be responsible for the day-to-day conduct of the trial. Their responsibilities include participant recruitment, data collection, data entry, data validation, monitoring data quality, data analysis, and communication with the coordinating centre in case of any issues or adverse events.

Other responsibilities of each participant site include:

- Monitoring the trial’s adherence to protocol, timelines, and overall objectives.
- Reviewing data related to safety and adverse events, and making recommendations regarding participant safety.
- Evaluating results from interim analyses and making recommendations for any necessary modifications to the trial.

### Composition of the data monitoring committee, its role and reporting structure {21a}

A Data Monitoring Committee has not been established for this trial, as the nature of the study does not warrant the need for an independent monitoring body. The study involves minimal risks to participants, and ongoing safety monitoring will be conducted by the study investigators. Any adverse events will be carefully documented and reviewed by the research team, and appropriate actions will be taken as necessary. Additionally, all procedures adhere to institutional and ethical guidelines, providing oversight throughout the trial duration.

### Adverse event reporting and harms {22}

Participants will be closely monitored during the trial with regular assessments scheduled at predefined intervals (e.g., baseline, post-intervention, and follow-up). Participants and their caregivers will be encouraged to report any adverse events or unintended effects related to the trial interventions. Each reported adverse event will be assessed for its severity, relationship to the trial intervention, and potential impact on participant safety. This will involve classifying adverse events into categories such as mild, moderate, severe, and life-threatening. All adverse events will be documented in the trial database, including details such as the type of event, date of occurrence, duration, and any actions taken (e.g., withdrawal from the study, medical intervention). Pursuant to EU Regulation 2017/745, MDCG 2020-10/1 Rev1 (Safety reporting in clinical investigation of medical devices under the Regulation EU 2017/745), Legislative Decree 46/97, Legislative Decree 507/92, Meddev Guideline 2.12−1 rev.8 and its supplement “Additional guidance regarding the vigilance system as outlined in MEDDEV 2.12-1 rev.8,” all incidents and serious adverse events that may occur during the investigation will be reported to Office V (Medical Device Vigilance) of the DGDFSC of the Italian Ministry of Health and the relevant Ethics Committee. Since this is a clinical study, serious adverse events may also be forwarded to Office VI of the Italian Ministry of Health for information. Participants experiencing adverse events will receive appropriate medical care and follow-up as needed.

Beyond general adverse event reporting, several potential risks specific to using the Agilik device have been identified. A possible risk is that the child may be assisted too much by the device, leading to reduced active engagement or decreased performance. This is mitigated by carefully adjusting torque parameters during the in-clinic phase, ensuring that the device supports the child without replacing their active effort or causing fatigue. Another potential risk is the possibility of falls or balance loss during training. To minimize this, participants are instructed to always use the device under the supervision of a caregiver. Training can take place both indoors and outdoors, depending on family preferences, but participants are advised to avoid walking in the rain or in any condition that may expose the device to moisture or increase the risk of slipping. In the event of an unexpected device shutdown while walking, caregiver supervision remains essential to ensure immediate support and prevent unsafe situations.

Technical issues with the device may also occur. To address this, the research team remains available throughout the intervention to provide timely assistance, and families are asked to promptly report any malfunction so that corrective actions can be taken. Another potential issue is reduced adherence to the training program, particularly during warmer months, when wearing the orthosis may cause discomfort due to heat or sweating, or when participants are already engaged in other daily activities, adding an additional burden to their routine. To support adherence and motivation, the research team maintains regular contact with families throughout the intervention and provides encouragement and practical guidance.

Finally, some participants or caregivers may initially feel apprehensive about managing the technology independently. To facilitate a smooth transition to home use, the last in-clinic sessions are dedicated to caregiver-led practice, allowing them to gain confidence and autonomy before beginning home training.

### Frequency and plans for auditing trial conduct {23}

No formal auditing process has been planned for the conduct of this trial. However, the trial will adhere to standard operating procedures and institutional guidelines to ensure compliance with ethical and regulatory requirements. Any issues related to trial conduct will be managed by the research team and reviewed as part of routine oversight.

### Plans for communicating important protocol amendments to relevant parties (e.g., trial participants, ethical committees) {25}

All investigators and research team members will be promptly informed of any changes to the protocol during team meetings and through official email notifications. Any modifications that affect eligibility criteria, outcomes, or analyses will be submitted to the Ethical Committee for review and approval prior to implementation. Participants will be informed of significant protocol changes through direct communication, ensuring they understand how these modifications may affect their participation in the trial. Updated consent forms will be provided for them to review and sign if necessary.

### Dissemination plans {31a}

The trial results will be communicated to various stakeholders through multiple channels. Upon conclusion of the study, findings will be published in peer-reviewed scientific journals and presented at relevant conferences to inform healthcare professionals and the wider scientific community. Additionally, if requested, results will be shared with participants and their families in a summarized and accessible format, ensuring transparency and engagement. The study outcomes will also be available under request in publicly accessible trial registries and results databases.

There are no publication restrictions, and the investigators commit to sharing the trial data openly, adhering to ethical guidelines. Any intellectual property arising from the study will be handled in accordance with institutional policies, without limiting the publication of findings.

## Discussion

This multicentre randomized controlled trial described in this protocol aims to evaluate the clinical effectiveness of the Agilik powered knee-ankle-foot orthosis in children with cerebral palsy and crouch gait across seven participating centres in Italy. One of the primary operational challenges will be ensuring consistency in data collection and intervention implementation across multiple sites. This will require frequent communication between the study centres and the central coordination team, as well as regular training for staff involved in assessments and interventions to ensure adherence to the study protocol. Differences in available resources or variations in site-specific practices could potentially affect the execution of the trial, so these will be actively monitored and adjusted as necessary.

Participant recruitment may also present challenges, especially in securing the required number of eligible children with CP exhibiting crouch gait across multiple centres. While multiple recruitment strategies will be employed to meet the sample size, there is always a risk of slower-than-expected enrolment, particularly if eligible participants are geographically dispersed or face barriers to participation such as travel constraints.

Another consideration is the need for ongoing support for participants and their families to encourage adherence to the intervention protocol. Given that the trial involves the use of specialized devices (i.e., Agilik orthosis), participants may encounter technical difficulties or discomfort, potentially affecting compliance. Addressing these issues promptly will be key to maintaining engagement throughout the trial.

Logistical issues may arise with the transportation of Agilik between clinical sites and participants’ homes. Ensuring that all necessary equipment is available and functional for each visit and that participants have the support needed to use the devices correctly will be a priority.

An important methodological consideration is that participants in both groups may continue their usual standard therapy, if already prescribed, and that the total training time may not be perfectly balanced between the two groups. However, standard therapy in the participating Italian regions is typically highly variable, ranging from absent or low intensity (0–2 sessions per week) to very intensive (up to 5 one-hour sessions per week). To account for potential differences in overall training exposure, all rehabilitation activities, including the type, frequency, and duration of standard therapy, will be systematically recorded for each participant. This will allow therapy dosage to be included as a covariate in the data analysis and will help disentangle the specific contribution of the Agilik intervention from that of routine care.

From a clinical perspective, another challenge may stem from the heterogeneity of crouch gait within the cerebral palsy population, which can arise from multiple underlying causes. To mitigate this variability and improve comparability between groups, randomization will be stratified by GMFCS level and gender, two variables strongly associated with gait function and global motor impairment. Detailed baseline clinical and instrumental measures (e.g., GMFM-88, 3D gait analysis, ROM, spasticity, and muscle length assessments) will also be collected to comprehensively characterize participants and to support appropriate adjustment in subsequent analyses. Given the heterogeneity of crouch gait and the multifactorial nature of motor impairments in cerebral palsy, results from this study may not be fully generalizable to the entire CP population. However, stratified randomization and detailed characterization of participants at baseline are expected to reduce variability and strengthen the interpretability of findings.

A limitation of the study is the absence of an objective, instrumented assessment of knee strength, which is an important factor in understanding crouch gait and its potential improvement. Its absence may limit the ability to directly quantify changes in muscular performance associated with the intervention. Future studies may benefit from incorporating an instrumented strength assessment to provide a more comprehensive evaluation of treatment effects.

In conclusion, this study protocol outlines a randomized controlled trial designed to evaluate the clinical efficacy, usability, and acceptability of the Agilik powered KAFO in children with CP and crouch gait. The trial aims to generate new evidence that could influence clinical practice and guide future interventions for managing crouch gait, with the ultimate goal of improving the quality of life for children with CP. Adopting a patient-centred approach, the study not only focuses on motor and functional outcomes but also considers the perspectives of patients and their families. Particular attention will be given to their experiences, including any difficulties or discomfort encountered during the intervention. The integration of quantitative and qualitative data will support the development of sustainable, home-based rehabilitation strategies and inform the broader implementation of personalized assistive technologies in paediatric neurorehabilitation.

## Trial status

The current version of the protocol is 1.0, dated 20 April 2024. Participant recruitment began in October 2024 and is expected to be completed by July 2026. The total duration of the study is 24 months; therefore, data collection will conclude in October 2026, and the results are expected by March 2027. Any future amendments to the protocol will be documented with updated version numbers and corresponding dates, as necessary.

## Supporting information

S1 File Clinical investigation plan. Overview of the clinical investigation plan, including study design, intervention phases, and assessment timeline.
(PDF)

## Abbreviations

6MWT: Six Minute Walking Test
10mWT: Ten Meter Walking Test
CP: Cerebral Palsy
COP: Centre of Pressure
COPM: Canadian Occupational Performance Measure
EMG: Electromyography
GMFCS: Gross Motor Function Classification System
GMFM-88: Gross Motor Function Measure-88
ITT: Intention-To-Treat
KAFO: Knee-Ankle-Foot Orthosis
KEMS: Knee Extension during Mid-Stance
MAS: Modified Ashworth Scale
PP: per-protocol
RCT: Randomized Controlled Trial
REDCap: Research Electronic Data Capture
ROM: Range of Motion
SUS: System Usability Scale
TAM-3: Technology Acceptance Model 3

## References

[1] Molnar GE. Rehabilitation in cerebral palsy. West J Med. 1991;154(5):569–72. 1866952 PMC1002833

[2] Yeargin-Allsopp M, Van Naarden Braun K, Doernberg NS, Benedict RE, Kirby RS, Durkin MS. Prevalence of cerebral palsy in 8-year-old children in three areas of the United States in 2002: a multisite collaboration. Pediatrics. 2008;121(3):547–54. doi: 10.1542/peds.2007-1270 18310204

[3] Binder H, Eng GD. Rehabilitation management of children with spastic diplegic cerebral palsy. Arch Phys Med Rehabil. 1989;70(6):482–9. doi: 10.1016/0003-9993(89)90012-9 2658915

[4] Perry J. Pathologic gait. Instr Course Lect. 1990;39:325–31. 2186119

[5] Rosenbaum PL, Russell DJ, Cadman DT, Gowland C, Jarvis S, Hardy S. Issues in measuring change in motor function in children with cerebral palsy: a special communication. Phys Ther. 1990;70(2):125–31. doi: 10.1093/ptj/70.2.125 2404286

[6] Bottos M, Gericke C. Ambulatory capacity in cerebral palsy: prognostic criteria and consequences for intervention. Dev Med Child Neurol. 2003;45(11):786–90. doi: 10.1017/s0012162203001452 14580136

[7] Olney SJ, MacPhail HE, Hedden DM, Boyce WF. Work and power in hemiplegic cerebral palsy gait. Phys Ther. 1990;70(7):431–8. doi: 10.1093/ptj/70.7.431 2356219

[8] Damiano DL, Kelly LE, Vaughn CL. Effects of quadriceps femoris muscle strengthening on crouch gait in children with spastic diplegia. Phys Ther. 1995;75(8):658–67; discussion 668-71. doi: 10.1093/ptj/75.8.658 7644570

[9] Wiley ME, Damiano DL. Lower-extremity strength profiles in spastic cerebral palsy. Dev Med Child Neurol. 1998;40(2):100–7. doi: 10.1111/j.1469-8749.1998.tb15369.x 9489498

[10] Tedroff K, van der Krogt M. Spasticity and dystonia consequences, management, and future perspectives; 2020. p. 77–93.

[11] Rogozinski BM, Davids JR, Davis RB 3rd, Jameson GG, Blackhurst DW. The efficacy of the floor-reaction ankle-foot orthosis in children with cerebral palsy. J Bone Joint Surg Am. 2009;91(10):2440–7. doi: 10.2106/JBJS.H.00965 19797580

[12] Damiano DL, DeJong SL. A systematic review of the effectiveness of treadmill training and body weight support in pediatric rehabilitation. J Neurol Phys Ther. 2009;33(1):27–44. doi: 10.1097/NPT.0b013e31819800e2 19265768 PMC2982788

[13] Beretta E, Storm FA, Strazzer S, Frascarelli F, Petrarca M, Colazza A, et al. Effect of robot-assisted gait training in a large population of children with motor impairment due to cerebral palsy or acquired brain injury. Arch Phys Med Rehabil. 2020;101(1):106–12. doi: 10.1016/j.apmr.2019.08.479 31562873

[14] Lerner ZF, Damiano DL, Bulea TC. The effects of exoskeleton assisted knee extension on lower-extremity gait kinematics, kinetics, and muscle activity in children with cerebral palsy. Sci Rep. 2017;7(1):13512. doi: 10.1038/s41598-017-13554-2 29044202 PMC5647342

[15] Dierwechter B, Kolakowsky-Hayner SA. Journey to 1 Million steps: a retrospective case series analyzing the implementation of robotic-assisted gait training into an outpatient pediatric clinic. Pediatr Phys Ther. 2024;36(2):285–93. doi: 10.1097/PEP.0000000000001097 38349640

[16] Smania N, Gandolfi M, Marconi V, Calanca A, Geroin C, Piazza S, et al. Applicability of a new robotic walking aid in a patient with cerebral palsy. Case report. Eur J Phys Rehabil Med. 2012;48(1):147–53. 22543558

[17] Tagoe EA, Fang Y, Williams JR, Stone JL, Lerner ZF. Exoskeleton gait training on real-world terrain improves spatiotemporal performance in cerebral palsy. Front Bioeng Biotechnol. 2024;12:1503050. doi: 10.3389/fbioe.2024.1503050 39741499 PMC11685018

[18] Lerner ZF, Damiano DL, Bulea TC. A lower-extremity exoskeleton improves knee extension in children with crouch gait from cerebral palsy. Sci Transl Med. 2017;9(404):eaam9145. doi: 10.1126/scitranslmed.aam9145 28835518 PMC9993999

[19] Bulea TC, Lerner ZF, Gravunder AJ, Damiano DL. Exergaming with a pediatric exoskeleton: Facilitating rehabilitation and research in children with cerebral palsy. IEEE Int Conf Rehabil Robot. 2017;2017:1087–93. doi: 10.1109/ICORR.2017.8009394 28813966 PMC10436702

[20] Bayón C, Martín-Lorenzo T, Moral-Saiz B, Ramírez Ó, Pérez-Somarriba Á, Lerma-Lara S, et al. A robot-based gait training therapy for pediatric population with cerebral palsy: goal setting, proposal and preliminary clinical implementation. J Neuroeng Rehabil. 2018;15(1):69. doi: 10.1186/s12984-018-0412-9 30053857 PMC6063005

[21] Hunt M, Everaert L, Brown M, Muraru L, Hatzidimitriadou E, Desloovere K. Effectiveness of robotic exoskeletons for improving gait in children with cerebral palsy: a systematic review. Gait Posture. 2022;98:343–54. doi: 10.1016/j.gaitpost.2022.09.082 36306544

[22] Basla C, Hungerbühler I, Meyer JT, Wolf P, Riener R, Xiloyannis M. Usability of an exosuit in domestic and community environments. J Neuroeng Rehabil. 2022;19(1):131. doi: 10.1186/s12984-022-01103-6 36457037 PMC9714034

[23] Shideler BL, Bulea TC, Chen J, Stanley CJ, Gravunder AJ, Damiano DL. Toward a hybrid exoskeleton for crouch gait in children with cerebral palsy: neuromuscular electrical stimulation for improved knee extension. J Neuroeng Rehabil. 2020;17(1):121. doi: 10.1186/s12984-020-00738-7 32883297 PMC7469320

[24] Devine TM, Alter KE, Damiano DL, Bulea TC. A randomized cross-over study protocol to evaluate long-term gait training with a pediatric robotic exoskeleton outside the clinical setting in children with movement disorders. PLoS One. 2024;19(7):e0304087. doi: 10.1371/journal.pone.0304087 38976710 PMC11230531

[25] Enright PL. The six-minute walk test. Respir Care. 2003;48(8):783–5. 12890299

[26] Guinet AL, Desailly E. Six-minute walk test (6MWT) in children with cerebral palsy. Systematic review and proposal of an adapted version. Ann Phys Rehabil Med. 2018;61:e304. doi: 10.1016/j.rehab.2018.05.1307

[27] Clarkson HM, Pace P. Valutazione cinesiologica. Esame della mobilità articolare e della forza muscolare. 2nd ed. Edi.Ermes srl; 2002.

[28] Herrero P, Carrera P, García E, Gómez-Trullén EM, Oliván-Blázquez B. Reliability of goniometric measurements in children with cerebral palsy: a comparative analysis of universal goniometer and electronic inclinometer. A pilot study. BMC Musculoskelet Disord. 2011;12:155. doi: 10.1186/1471-2474-12-155 21740600 PMC3160434

[29] Morris S. Ashworth and Tardieu scales: their clinical relevance for measuring spasticity in adult and paediatric neurological populations. Phys Ther Rev. 2002;7:53–62. doi: 10.1179/108331902125001770

[30] Horie S, Ansari B, Masterson C, Devaney J, Scully M, O’Toole D, et al. Hypercapnic acidosis attenuates pulmonary epithelial stretch-induced injury via inhibition of the canonical NF-κB pathway. Intensive Care Med Exp. 2016;4(1):8. doi: 10.1186/s40635-016-0081-6 27001525 PMC4801837

[31] Russell DJ, Rosenbaum PL, Avery LM, Lane M. Gross Motor Function Measure (GMFM-66 e GMFM-88) - Manuale dell’utente. In: Stefanoni G, editor. Armando Editore; 2006.

[32] Nordmark E, Hägglund G, Jarnlo GB. Reliability of the gross motor function measure in cerebral palsy. Scand J Rehabil Med. 1997;29(1):25–8. doi: 10.2340/1650197719972528 9084102

[33] Santos M, Zdravevski E, Albuquerque C, Coelho PJ, Pires IM. Ten Meter Walk Test for motor function assessment with technological devices based on lower members’ movements: a systematic review. Comput Biol Med. 2025;187:109734. doi: 10.1016/j.compbiomed.2025.109734 39904103

[34] Graser JV, Letsch C, van Hedel HJA. Reliability of timed walking tests and temporo-spatial gait parameters in youths with neurological gait disorders. BMC Neurol. 2016;16:15. doi: 10.1186/s12883-016-0538-y 26830919 PMC4736644

[35] Souissi H, Zory R, Bredin J, Gerus P. Comparison of methodologies to assess muscle co-contraction during gait. J Biomech. 2017;57:141–5. doi: 10.1016/j.jbiomech.2017.03.029 28433389

[36] Vinti M, Bayle N, Merlo A, Authier G, Pesenti S, Jouve J-L, et al. Muscle shortening and spastic cocontraction in gastrocnemius medialis and peroneus longus in very young hemiparetic children. Biomed Res Int. 2018;2018:2328601. doi: 10.1155/2018/2328601 29951529 PMC5987331

[37] Villani M, Avaltroni P, Scordo G, Rubeca D, Kreynin P, Bereziy E, et al. Evaluation of EMG patterns in children during assisted walking in the exoskeleton. Front Neurosci. 2024;18:1461323. doi: 10.3389/fnins.2024.1461323 39513047 PMC11541598

[38] Davis RB, Õunpuu S, Tyburski D, Gage JR. A gait analysis data collection and reduction technique. Hum Mov Sci. 1991;10:575–87. doi: 10.1016/0167-9457(91)90046-Z

[39] Ferrari A, Benedetti MG, Pavan E, Frigo C, Bettinelli D, Rabuffetti M, et al. Quantitative comparison of five current protocols in gait analysis. Gait Posture. 2008;28(2):207–16. doi: 10.1016/j.gaitpost.2007.11.009 18206374

[40] Rabuffetti M, Marzegan A, Crippa A, Carpinella I, Lencioni T, Castagna A, et al. The LAMB gait analysis protocol: definition and experimental assessment of operator-related variability. Proc Inst Mech Eng H. 2019;233(3):342–53. doi: 10.1177/0954411919827033 30706762

[41] Kainz H, Graham D, Edwards J, Walsh HPJ, Maine S, Boyd RN, et al. Reliability of four models for clinical gait analysis. Gait Posture. 2017;54:325–31. doi: 10.1016/j.gaitpost.2017.04.001 28411552

[42] Ruhe A, Fejer R, Walker B. Center of pressure excursion as a measure of balance performance in patients with non-specific low back pain compared to healthy controls: a systematic review of the literature. Eur Spine J. 2011;20(3):358–68. doi: 10.1007/s00586-010-1543-2 20721676 PMC3048236

[43] Carswell A, McColl MA, Baptiste S, Law M, Polatajko H, Pollock N. The Canadian Occupational Performance Measure: a research and clinical literature review. Can J Occup Ther. 2004;71(4):210–22. doi: 10.1177/000841740407100406 15586853

[44] Kang M, Smith E, Goldsmith CH, Switzer L, Rosenbaum P, Wright FV, et al. Documenting change with the Canadian Occupational Performance Measure for children with cerebral palsy. Dev Med Child Neurol. 2020;62(10):1154–60. doi: 10.1111/dmcn.14569 32491226

[45] Brooke J. SUS: A quick and dirty usability scale. Usability Eval Ind. 1995;189.

[46] Peres SC, Pham T, Phillips R. Validation of the System Usability Scale (SUS). Proc Hum Factors Ergon Soc Annu Meet. 2013;57(1):192–6. doi: 10.1177/1541931213571043

[47] Venkatesh V, Bala H. Technology acceptance model 3 and a research agenda on interventions. Decis Sci. 2008;39:273–315. doi: 10.1111/j.1540-5915.2008.00192.x

[48] Harris PA, Taylor R, Thielke R, Payne J, Gonzalez N, Conde JG. Research electronic data capture (REDCap)--a metadata-driven methodology and workflow process for providing translational research informatics support. J Biomed Inform. 2009;42(2):377–81. doi: 10.1016/j.jbi.2008.08.010 18929686 PMC2700030

[49] Harris PA, Taylor R, Minor BL, Elliott V, Fernandez M, O’Neal L, et al. The REDCap consortium: building an international community of software platform partners. J Biomed Inform. 2019;95:103208. doi: 10.1016/j.jbi.2019.103208 31078660 PMC7254481

[50] Storm FA, Petrarca M, Beretta E, Strazzer S, Piccinini L, Maghini C, et al. Minimum clinically important difference of gross motor function and gait endurance in children with motor impairment: a comparison of distribution-based approaches. Biomed Res Int. 2020;2020:2794036. doi: 10.1155/2020/2794036 32509855 PMC7246400

[51] Bonnefoy-Mazure A, Sagawa Y Jr, Lascombes P, De Coulon G, Armand S. Identification of gait patterns in individuals with cerebral palsy using multiple correspondence analysis. Res Dev Disabil. 2013;34(9):2684–93. doi: 10.1016/j.ridd.2013.05.002 23770664

[52] Sozu T, Sugimoto T, Hamasaki T, Evans SR. Sample size determination in clinical trials with multiple endpoints. Springer Heidelberg; 2015.

