# Supplementary material for: Agilik@home: A randomized controlled trial protocol to evaluate the effects of home-based training with the Agilik powered KAFO in children with cerebral palsy and crouch gait
Source: PLoS One. 2026 Mar 25;21(3):e0340877. doi: 10.1371/journal.pone.0340877 (PMC13016275; doi:10.1371/journal.pone.0340877)
Supplement: S1 File — Overview of the clinical investigation plan, including study design, intervention phases, and assessment timeline. (PDF) [file pone.0340877.s001.pdf]

CLINICAL INVESTIGATION PLAN
According to MDR 2017/745

General Information

TITLE:	Agilik@home: demonstrate the clinical improvement due to the home use of Agilik in children with Cerebral Palsy
STUDY CODE:	Id.1122
VERSION:	Version n.1.0 of the 20.04.2024
PREVIOUS VERSIONS:	NA
SPONSOR:	IRCCS Eugenio Medea – sezione scientifica dell'Associazione "La Nostra Famiglia" – Sede legale in Via don Luigi Monza, 1 - 22037 Ponte Lambro (CO)
CONTACT PERSON OR LEGAL REPRESENTATIVE OF THE SPONSOR:	Dott.ssa Luisa Minoli – Legal Representative of Eugenio Medea – sezione scientifica dell'Associazione "La Nostra Famiglia"<br><br>Contact person: Emilia Biffi, PhD, Biomedical Engineer
MANUFACTURER:	**MANUFACTURER:**<br>Bionic Power Inc.<br>2661 Lillooet Street, Vancouver, BC, V5M 4P7, Canada<br>Tel: +1.778.729.0680<br>Email: info@bionic-power.com<br>Website: https://www.bionic-power.com/<br><br>**DISTRIBUTOR:**<br>ORTHOSERVICE RO+TEN srl<br>Headquarter: ORTHOSERVICE AG · Via Milano 7 · CH-6830 Chiasso (TI) · Switzerland<br>Tel. 0041 (0) 91 822 00 88, Fax 0041 (0) 91 822 00 89<br>Email: info@orthoservice.com - www.orthoservice.com
FUNDING:	The protocol is part of the project Fit4MedRob- Piano nazionale per gli investimenti complementari (PNC) funded by Ministry of University and Research.

PARTICIPANTS CENTRES

MAIN CLINICAL CENTRE	IRCCS E. MEDEA – Associazione "La Nostra Famiglia"<br>Via Don Luigi Monza, 20

	23842 Bosisio Parini (LC) – Italia<br>Tel. +39 031 877.111, Fax +39 031 877.499<br><br>Responsabile Scientifico:<br>Emilia Biffi, PhD, Biomedical Engineer<br>Via Don Luigi Monza, 20<br>23842 Bosisio Parini (LC) – Italia<br>Tel. +39 031 877.862<br>Email: emilia.biffi@lanostrafamiglia.it<br><br>Responsabile Clinico:<br>Cristina Maghini, MD<br>Via Don Luigi Monza, 20<br>23842 Bosisio Parini (LC) – Italia<br>Tel. +39 031 877.862<br>Email: cristina.maghini@lanostrafamiglia.it
CLINICAL CENTRE #2	IRCCS Fondazione Stella Maris<br>Viale del Tirreno 341/ ABC<br>56128 Calambrone (Pisa) – Italia<br>Tel. 050 886284<br><br>Sperimentatore Principale:<br>Prof.ssa Giuseppina Sgandurra, MD, PhD<br>IRCCS Fondazione Stella Maris<br>Viale del Tirreno 341/ ABC<br>56128 Calambrone (Pisa) – Italia<br>Tel. 050 886284<br>Email: giuseppina.sgandurra@fsm.unipi.it
CLINICAL CENTRE #3	Fondazione Don Carlo Gnocchi Onlus<br>Via Maresciallo Caviglia, 30<br>00194 Roma<br>e<br>Via Casal del Marmo 401<br>00166 Roma<br><br>Sperimentatore principale:<br>Dr. Laura Iuvone, MD<br>Responsabile Servizio di Riabilitazione Infantile<br>Fondazione Don Carlo Gnocchi Onlus<br>Via Maresciallo Caviglia, 30<br>00194 Roma<br>Tel 06/330861<br>mail: liuvone@dongnocchi.it

CLINICAL CENTRE #4	Fondazione Mondino<br>Via Mondino, 2 - 27100 Pavia<br><br>Sperimentatore Principale:<br>Dr.ssa Sabrina Signorini<br>Fondazione Mondino<br>Via Mondino, 2 - 27100 Pavia<br>Email: sabrina.signorini@mondino.it
CLINICAL CENTRE #5	Centro S. Maria al Mare - Fondazione Don Carlo Gnocchi<br>Via Leucosia, 16, 84131 Salerno SA<br><br>Sperimentatore Principale:<br>Dr. Irene Aprile<br>Centro S. Maria al Mare - Fondazione Don Carlo Gnocchi<br>Via Leucosia, 16, 84131 Salerno SA<br>Email: iaprile@dongnocchi.it
CLINICAL CENTRE #6	Fondazione Don Carlo Gnocchi - IRCCS S. Maria Nascente<br>Via Alfonso Capecelatro, 66, 20148 Milano MI<br><br>Sperimentatore Principale:<br>Dr.ssa Angela Cavalagli<br>Fondazione Don Carlo Gnocchi - IRCCS S. Maria Nascente<br>Via Alfonso Capecelatro, 66, 20148 Milano MI<br>Email: acavalagli@dongnocchi.it

INFORMAZIONI DI CONTATTO

CONTACT FOR THE VIGILANCE DEVICE:	Ing. Paola Grigioni<br>Email: paola.grigioni@lanostrafamiglia.it<br><br>Dr. Giuseppe Paladino<br>Email:dirsan@fsm.unipi.it<br><br>Dr.ssa Irene Aprile<br>Email: iaprile@dongnocchi.it<br><br>Dr.ssa Luisa Gervasio<br>Email: luisa.gervasio@mondino.it<br><br>Dott.ssa Leone Maria Rosaria<br>Email: lrosaria@dongnocchi.it<br><br>Dott. Mauro De Chiara<br>Email:mdechiara@dongnocchi.it

PROTOCOL APPROVAL

The Investigators:

- approve the present Protocol;
- declare that the study will be conducted in accordance with the provisions of this Protocol.

DATE	NAME OF THE INVESTIGATOR
22.04.2024	Emilia Biffi
22.04.2024	Cristina Maghini
22.04.2024	Giuseppina Sgandurra
22.04.2024	Laura Iuvone
22.04.2024	Sabrina Giovanna Signorini
22.04.2024	Irene Aprile
22.04.2024	Dr.ssa Angela Cavalagli

Index

General Information 1
Synopsis of the clinical investigation 7
Device information 8
Device identification 8
Device description 8
Intended purpose of the device and target population 9
Manufacturer 9
Traceability 10
Materials coming into contact with the human body 10
Medical or surgical procedures involved in its use 10
Necessary training and experience for the use of the device 10
Background literature review 10
Current state of the art in clinical care in the relevant field of application and the proposed benefits of the new device 11
Risks and clinical benefits 11
Description of the relevance of the clinical investigation in the context of the state of the art of clinical practice 14
Objectives and hypotheses of the clinical investigation 14
Design of the clinical investigation 14
Duration 14
General Information of the protocol 14
Endpoints and measured variables 15
Information on the investigational device, on any comparator and on any other device or medication to be used in the clinical investigation 16
Information on subjects 16
Measures to be taken to minimise bias and management of potential confounding factors 17
Procedures 17
Monitoring plan 18
Enrolment 18
Statistical considerations 18
Data management 18
Intellectual property 19
Information about any amendments to the study 19
Policy regarding follow-up and management of any deviations from the protocol 19
Accountability regarding the device 19
Ethical considerations 20
Description of the Informed consent process 20
Safety reporting 20
Criteria and procedures for follow-up of subjects following the end, temporary halt or early termination of an investigation, for follow-up of subjects who have withdrawn their consent and procedures for subjects lost to follow-up 20

Description of the arrangements for taking care of the subjects after their participation in the clinical investigation has ended 20
Policy for data publication 21
List of the technical and functional features of the device 21
Bibliography 21

Synopsis of the clinical investigation

The purpose of this post market study is to demonstrate the clinical improvement due to the use of a powered extension assist knee ankle foot orthosis, or EA-KAFO, in individuals with knee extension deficiency due to cerebral palsy (CP) in a domiciliary setting. The EA-KAFO tested in this investigation, whose commercial name is Agilik™, is registered as a Class 1 medical device in the EU MDR and with the US FDA (Food and Drugs Administration, USA). The design of the study is schematized in Figure 1.

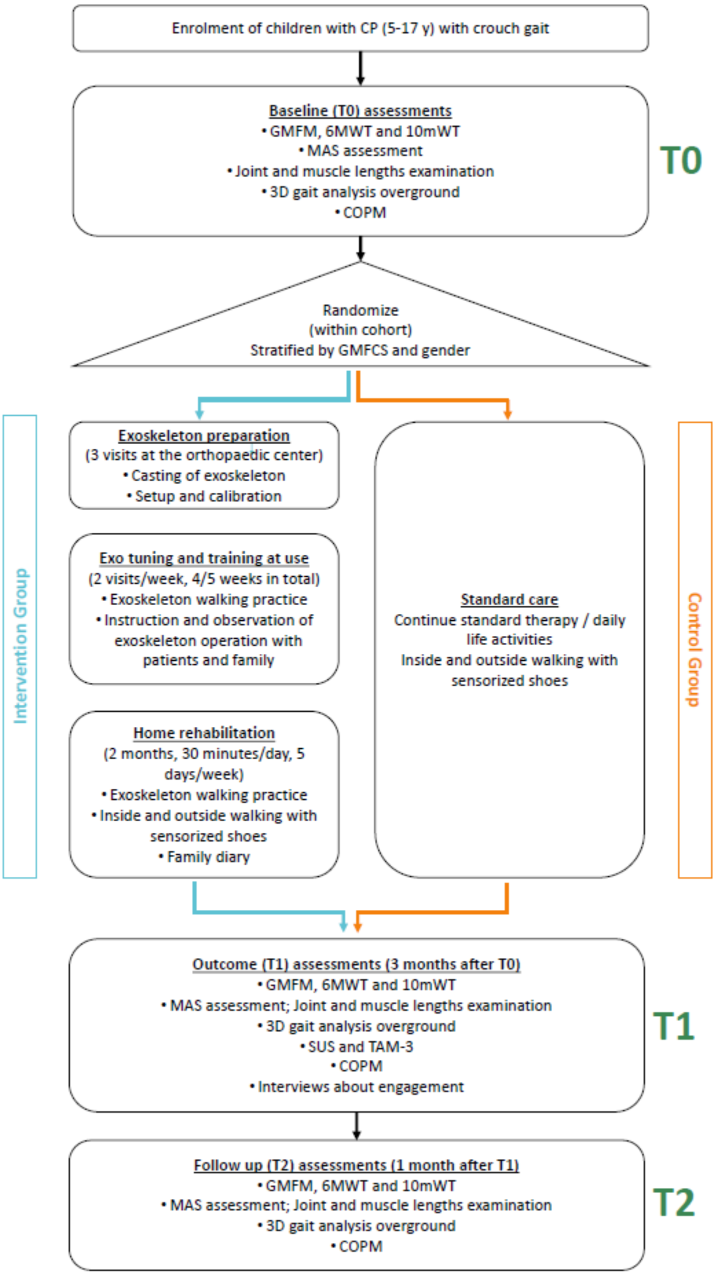

Figure 1: study design

Device information

Device identification

Agilik™

Device description

Design

The fabricated knee-ankle-foot orthosis (KAFO) is individually developed for each patient. For this reason, the illustrations in the Instruction for Use attached to the protocol ("D0476-04_IFU AGILIK MVP Lite) only show one possible (KAFO) that has been fitted with the "Agilik."

Function

The Agilik is a powered orthosis system that can assist or resist motion independently in each gait phase. It is normally used as a pair of joint orthoses, one for each leg. The device can apply up to 12 Nm across the knee joint in the direction of either flexion or extension. The system consists of an orthosis for each leg, a battery, a carry pack, cabling and application software running on a computer. The orthosis itself consists of an electro-mechanical actuator with integrated electronics that is attached as a lateral hinge on a conventional, patient-specific custom-molded KAFO. A foot pressure sensor is embedded in the footbed of the KAFO and connected to the actuator.
The foot sensor and angular velocity are used to detect the gait phase of the patient and communicate with the motor controller to provide a unique torque for each gait phase. The torque ramp and intensity can be modified through the Agilik App to cater towards individual patients by assisting or resisting motion during their gait. This allows for the clinician working with the patient to adjust the settings of the device for the patient's needs.

Classification

The Agilik is classified as a Class 1 medical device. According to IEC 60601-1 the Agilik system is classified as a body-worn INTERNALLY POWERED MEDICAL EQUIPMENT rated for NON-CONTINUOUS OPERATION. The normal duty cycle of the device is regular use for one full charge of the battery, and then recharging the battery (~3.5 hours) before use of the device again.

Applied Parts

- Battery pack
- KAFO shell and straps

No other applied parts or parts subject to the requirements of applied parts are identified through the risk management process.

Enclosures

Any enclosures rated for protection against ingress of fluid and particulate matter are marked accordingly using IPXX format on the equipment. See the Instruction for Use attached to this protocol (D0476-18_IFU AGILIK 1.1) for a description of relevant markings.

Intended purpose of the device and target population

The product is intended for orthotic fittings of the lower limbs. The product is intended for patients with lower extremity weakness resulting in gait pathology such as, but not limited to crouch gait from

a diagnosis of cerebral palsy, muscular dystrophy, spina bifida, or incomplete spinal cord injury or post stroke hemiparesis.

Conditions of Use:

- The product is intended for forward walking on flat, dry ground. Adult supervision is required for children.
- It is the responsibility of the therapist/clinician to determine if the patient is physically and cognitively able to understand and use the product.
- If the patient is dependent upon existing patient support systems like crutches or walkers, it is recommended to continue use of them alongside the Agilik. Any walker used with the Agilik must be adjusted wide enough to accommodate the KAFO plus actuator width.
- The product can be used for home and community mobility at the discretion of the clinician considering the ability of the patient to use the device. In this case, the patient and family must be trained by a Bionic Power certified individual.
- The product must not be used for other activities including, but not limited to, running, jumping, squatting, and ascending or descending stairs/ramps. If the patient needs to do any movement or enter any terrain not intended for use with the Agilik, the device should be powered down or removed.
- The product must only be used with the correct customised KAFO developed by an authorised orthotist for each specific patient with the patients' shoes worn on the outside of the KAFO.
- Fitting of a patient with the product and KAFO may only be carried out by a Bionic Power certified clinician.
- The product is intended exclusively for multiple use by a single patient. Use of the product by more than one patient is not approved by the manufacturer.

Manufacturer

Bionic Power Inc.
2661 Lillooet Street, Vancouver, BC, V5M 4P7, Canada
Tel: +1.778.729.0680
Email: info@bionic-power.com
Website: https://www.bionic-power.com/

Traceability

The Agilik orthosis used during the investigation will bear a label to uniquely identify them, including a serial number.

Materials coming into contact with the human body

The Agilik is a robotic knee hinge integrated in a custom KAFO; the Agilik itself is not in direct contact with the skin or with the human body. For further information, see the Instruction for Use attached to the protocol (D0476-18_IFU AGILIK 1.1).

Medical or surgical procedures involved in its use

Adequately trained healthcare personnel will make patients wear and use the orthosis.

Necessary training and experience for the use of the device

Training and Qualification of the O&P Professional

The fitting of a patient KAFO shell with the product may only be carried out by certified O&P professionals who have been authorised with the corresponding Bionic Power training.

Training and Qualifications of the Therapist or Nursing Staff

Certified physical therapists and nursing staff may only handle and operate the product after being trained. Training must be carried out by Bionic Power Inc. authorised personnel.

Background literature review

Cerebral palsy (CP) is the most common motor disorder affecting children [1]. Among CP, spastic diplegia is very prevalent and occurs in more than 50% of the total CP population [2]. The motor deficits in spastic diplegia typically produce a walking pattern, termed crouch gait, which is characterized by exaggerated stance phase knee flexion, and may be accompanied by increased hip adduction and internal rotation, and a plantarflexed, neutral, or dorsiflexed ankle [3,4]. The majority of children with spastic diplegia eventually walk; however, this is often delayed and the walking patterns they develop differ from normal pediatric gait [5]. Even though CP is not considered a "progressive" disorder from a neurological standpoint, it has been reported that approximately 50% of those with CP who are ambulating as adolescents will cease to do so in early or middle adulthood due to worsening musculoskeletal deformities such as crouch gait [6], along with the secondary effects of reduced physical activity such as decreased strength and endurance.

There are multiple potential contributors to crouch gait, including muscle spasticity, contracture, poor selective motor control, dystonia, muscle imbalance, and muscle weakness. These symptoms can occur in muscles crossing the ankle, knee, and/or hip joints and involvement at any one or more of these joints may precipitate crouch. The relative contribution of each of these factors may vary across patients. The only potentially effective treatment when crouch gait is caused by muscle contracture is orthopedic surgery to increase muscle-tendon length. These procedures must be done advisably because muscles could become over-lengthened or weakened further leading to an exacerbation of crouch rather than correction[7]. Surgical outcomes have therefore been shown to be variable, but are purportedly better if the surgery is based on results from an instrumented gait analysis evaluation, simultaneous multi-level correction is performed when it is evident that multiple joints are involved, and procedures shown to cause excessive weakness are avoided[8].

Anti-gravity (extensor) muscle weakness in CP may not be the precipitating factor in crouch gait in the majority of cases, but clearly contributes as greater deterioration in crouch gait is observed with increasing age in part because the increase in muscle strength does not keep pace with the rate of body growth [9]. The major muscle groups that provide antigravity lower limb extension are the hip extensors, knee extensors, and ankle plantar flexors, all of which have been shown to be weak in children with CP [10]. Achieving and maintaining adequate strength levels in those with motor disabilities is challenging. Traditional ankle bracing compensates for weakness or spasticity by providing passive support or blocking unwanted motions [11], respectively, and often leads to greater weakness in these muscles over time. Clearly there is a need for more effective training strategies or devices that can preserve or possibly even augment strength on a continuous basis for those with CP, and thereby help to maintain gait function.

In order to improve crouch gait in children with CP, the Bionic Power Inc. has developed and commercialised Agilik™, whose effectiveness has already been demonstrated in a small sample of patients with CP (the device is CE-marked and FDA-approved)[12]. Therefore, the goal of this investigation is to demonstrate the clinical improvement due to the use of the device in home setting through a randomised study stratified according to gender and GMFCS.

Current state of the art in clinical care in the relevant field of application and the proposed benefits of the new device

Over the past several decades, mechanical and electrical devices have been used for gait training of patients with neurological disorders. Body-weight-supported treadmill systems (BWST) have shown varying degrees of effectiveness in gait rehabilitation for children with CP[13]. While the advanced robotic exoskeleton with BWST system (e.g. LOKOMAT by Hocoma Inc.) provides a safe and convenient environment for gait training[14], there is a paucity of wearable exoskeletons for the developmental age preventing from rehabilitation in domiciliary and ecological setting. Recently, few pilot studies demonstrated that overground gait training with wearable exoskeletons in hospital settings is effective in improving gait quality in small groups of children with CP[15, 16]. Other researchers tested an exoskeleton with weight-bearing on children with different diagnosis, demonstrating an increase in the number of steps performed[17]. Furthermore, a case study reported applicability of a robotic-assisted walker for helping a child with quadriplegic CP to walk overground[18]. Finally, a recent review highlighted that robotic exoskeletons have the potential to improve the mobility of CP children and to increase community participation and quality of life and suggest to perform works involving larger controlled intervention studies[19].

Despite these preliminary works there is a paucity of options and of biomechanical data supporting exoskeleton effectiveness. The Bionic Power Inc. proposes Agilik as a potential solution for gait training of children with crouch gait. Owing to the heterogeneous causes of crouch, they developed a device in which the type of assistance can be optimised and personalised for the individual user. This enhances its potential as a rehabilitative device compared to existing systems.

Risks and clinical benefits

Anticipated Benefit:

Participants to the study could benefit in using the device since the Agilik aids the user in standing, improves walking pattern and reduces crouch and energy expenditure, thus improving gait endurance.

Classification of Risk (for the study as a whole):

This study is classified as 45 CFR 46.404 - Research not involving greater than minimal risk to the children. The risks associated with this study are reasonable in relation to the anticipated benefit.

Specific Risks:

Motion capture: There is a slight risk of falling during the walking tasks, but these should be no greater than those encountered in everyday life. A trained physical therapist will be present at all times during data collection to ensure safety and assist subjects.

Specific risks related to the use of Agilik: Known and predictable risks related to the use of the Agilik are reported and described in the IFU from the manufacturer (section 4 of D0476-18_IFU AGILIK 1.1).

Description of the relevance of the clinical investigation in the context of the state of the art of clinical practice

Wearable devices that provide some type of external assistance and/or support to help a person perform a functional task, often referred to as robotic exoskeletons, are increasingly available as training/assistive devices. However, there is still a lack of evidence supporting their use and for determining optimal methods for specific patient populations.
This study proposes to demonstrate the clinical improvement due to the use of Agilik by Bionic Power in domiciliary setting, for patients with crouch gait due to CP.

Objectives and hypotheses of the clinical investigation

The primary purpose of this study is to improve the clinical use of a powered extension assist knee ankle foot orthosis (Agilik) in individuals with knee extension deficiency due to cerebral palsy in a domiciliary setting. Considering the preliminary data available, we will specifically focus on improvement of anti-gravity knee extension in mid stance (KEMS) and endurance in patients with crouch gait due to CP. Therefore, the primary aim is to compare the KEMS and the endurance at baseline, after 2 months of Agilik training (intervention group) or standard care/daily life activities (control group) and after one month of follow-up.
The secondary aim is to compare the two groups in terms of joint ROM and muscle length, spasticity, postural and motor abilities, gait speed and pattern and self-perceived performance.
Finally, the third aim is to evaluate the usability and the acceptability of the exoskeleton within the intervention group. Also some evidence about the user experience with the technology will be collected, encompassing the subjective perspective of both the patient and the caregivers.

Design of the clinical investigation

Duration

The duration of the investigation will be 24 months. The duration of the study for a single subject will be approximately 6 months.

General Information of the protocol

The clinical investigation will be a study with the following characteristics:

- Confirmatory and Not first-in-human;
- Randomised controlled study;
- Prospective study;
- Multicentric.

Endpoints and measured variables

In order to demonstrate the clinical improvement due to the use of the device, we will consider the following primary endpoints:

- Distance walked during a 6-minutes walking test (6MWT)[1];
- The KEMS measured during gait analysis assessment

[1] Enright, Paul L. "The six-minute walk test." Respiratory care 48.8 (2003): 783-785

In addition, secondary endpoints will be considered with the following purposes:

- at T0 (baseline), T1 (post intervention) and at T2 (follow-up)
    - Measurements of the Joint ROM and muscle lengths[2]
    - To determine the level of spasticity in the lower limbs with the Modified Ashworth scale (MAS)[3]
    - To assess postural and motor abilities with Gross Motor Function Measure-88 (GMFM-88)[4]
    - To assess functional gait assessment (walking speed) with the 10-meter walking test (10mWT)[5]
    - To evaluate kinematics and kinetics assessments (gait pattern) with the 3D gait analysis overground with EMG data acquisition
    - To determinate self-perception of performance in everyday living with Canadian Occupational Performance Measure (COPM)[6]
    - To quantify static balance with Centre of Pressure (COP) detection using the force platforms of the gait analysis lab when available
- at T1 only in the Intervention Group:
    - to assess the usability and acceptability of Agilik in parents/caregivers with the System Usability Scale (SUS)[7] and the Technology Acceptance Method 3 (TAM-3)[8]
    - to investigate aspects of behavioural engagement during the use of the Agilik device at home with semi-structured interviews with patients and their families

Only parents/caregivers of participants in the intervention group will be provided with a diary to freely explore, during the training, their thoughts and feelings about the device, in order to have a more extensive monitoring of the treatment.

Finally, an exploratory outcome will be:

- to perform a remote monitoring of gait behavior with sensorized shoes by Magnes (MAGNES AG, Hardturmstrasse 253, 8005 Zurich). If possible, during the months between T0 and T1 we will ask the participants of both groups to wear the shoes during indoor and outdoor walking, allowing us to acquire gait data to assess spatiotemporal parameters during daily life.

---

[2] Hazel M. Clarkson, Gail B. Gilewich. Valutazione cinesiologica. Esame della mobilità articolare e della forza muscolare. Edi ermes

[3] Morris S. Ashworth and Tardieu Scales: Their clinical relevance for measuring spasticity in adult and paediatric neurological populations. Physical Therapy Reviews. 2002 Mar 1;7(1):53-62.

[4] Gross Motor Function Measure (GMFM-66 e GMFM-88) - Manuale dell'utente. Dianne J. Russell – Peter L. Rosenbaum – Lisa M. Avery – Mary Lane. A cura di Giuseppe Stefanoni

[5] Copyright Academy of Neurologic Physical Therapy, "The ten-meter walk test." Academy of Neurological Physical Therapy, 2018; 42(2):174-220

[6] Carswell A, McColl MA, Baptiste S, Law M, Polatajko H, Pollock N. The Canadian Occupational Performance Measure: A Research and Clinical Literature Review. Canadian Journal of Occupational Therapy. 2004;71(4):210-222. doi:10.1177/000841740407100406

[7] J. Brooke, “SUS: A quick and dirty usability scale,” *Usability Eval. Ind.*, vol. 189, Nov. 1995.

[8] V. Venkatesh and H. Bala, “Technology Acceptance Model 3 and a Research Agenda on Interventions,” *Decis. Sci.*, vol. 39, no. 2, pp. 273–315, May 2008, doi: https://doi.org/10.1111/j.1540-5915.2008.00192.x.

The assessments will be collected using REDCap (Research Electronic Data Capture). REDCap is a secure, web-based software platform designed to support data capture for research studies, providing 1) an intuitive interface for validated data capture; 2) audit trails for tracking data manipulation and export procedures; 3) automated export procedures for seamless data downloads to common statistical packages; and 4) procedures for data integration and interoperability with external sources[20].

Information on the investigational device, on any comparator and on any other device or medication to be used in the clinical investigation

During the investigation, different sets of Agilik devices will be used for each of the six clinical experimental centres. They will be uniquely identified through labels reporting their serial numbers. No other devices will be tested. For other information on Agilik, refer to the section "*Device description*".

Information on subjects

We will enroll 40 children with knee extension deficiency due to CP according to sample size calculation.

Inclusion criteria:

- Volunteer who have a diagnosis of CP and a crouch gait
- Provision of signed and dated informed consent form.
- Stated willingness to comply with all study procedures and availability for the duration of the study, or alternatively, ability to do so based on parent report and physician observation during history and physical examination.
- Age between 5 and 17
- body weight between 20 and 125 Kg
- Knee flexion retraction assessed in supine position by less than 10°. Hamstring contracture as assessed by straight leg raising test does not limit ability to participate in the study.
- Subjects must not have had a tibio-tarsal arthrodesis. In addition, they must have at least 5° of passive dorsi-flexion of the ankle.
- Able to walk at least 3 m without stopping, with or without a walking aid.
- Able to understand and follow simple directions based on parent report and physician observation during historical and physical examination.
- GMFCS level I, II and III
- MAS score ≤ 2

Exclusion criteria:

- Any severe neurological, musculoskeletal and/or cardiorespiratory involvement preventing the ability to walk;
- A history of uncontrolled seizure in the past year
- Severe spasticity
- Hip and/or knee flexion contracture of more than 20°

Each subject is considered enrolled in the study when he/she (or his/her legal tutor) consents to participate by signing the informed consent. The recruitment will take place at IRCCS Medea, IRCCS

Fondazione Stella Maris, Fondazione Don Carlo Gnocchi Onlus Rome, Fondazione Mondino, Centro S. Maria al Mare - Fondazione Don Carlo Gnocchi and Fondazione Don Carlo Gnocchi - IRCCS S. Maria Nascente.

Measures to be taken to minimise bias and management of potential confounding factors

Participants to the study will be randomly divided into two groups in order to increase the likelihood that other variables, not taken into account in the study design, are evenly distributed within the two groups (randomised study).
All the procedures will be standardised to ensure repeatability of the collected measures and minimise operators' errors. The devices used to assess gait analysis and balance will be calibrated to ensure measurement repeatability.

Procedures

This is a study of subjects with CP to demonstrate the clinical improvement due to the use of Agilik in children with knee extension deficiency due to cerebral palsy (see Figure 1 in section *Synopsis of the clinical investigation)*. Participants will be enrolled based on inclusion and exclusion criteria and medical/physical history. Included children will be randomly divided into two groups (hereafter Intervention and Control Groups), stratified according to GMFM and gender, for a randomised control study. Both groups will start signing the informed consent.

The first visit is for physical examination and T0 assessments, which will include:

- 6MWT and KEMS measured during gait analysis, for demonstrating the clinical improvement due to the use of the device
- GMFM for postural and motor assessment
- 10mWT for functional gait assessment
- MAS to evaluate the level of spasticity in the lower limbs
- Joint ROM and muscle lengths examination performed manually by a medically responsible individual
- 3D gait analysis overground with EMG
- COPM for self-perceived performance assessment
- COP detection to quantify static balance.

Then, the Control Group will continue with standard therapy and daily life activities, and we will ask the participants to wear the sensorized shoes by Magnes during their outside walking when possible.

On the other hand, the Intervention group will start the intervention, which consists of:

1. 3 visits dedicated to exoskeleton preparation at the orthopedic center (casting for brace fabrication, set-up and calibration of the system).
2. Training with Agilik (4-5 weeks, 2 sessions/week) during which patients will practice walking with the device. Also the families will be trained to be able to autonomously use the system during home rehabilitation.
3. Home rehabilitation (2 months, 30 minutes/day, 5 days/week) during which subjects will practice walking with the device at home. Moreover, we will ask the participants to wear the sensorized shoes by Magnes during their outside walking when possible. Furthermore, children/parents will be asked to fill a diary to assess engagement in using this device.

Finally, 3 months after T0, both groups will undergo the outcome assessments (T1), and the follow-up assessments (T2) one month after T1. The follow-up assessment coincides with T0 assessments, while outcome assessments add, only for the Intervention Group, usability and acceptability evaluation of Agilik (SUS and TAM-3). Semi-structured interviews with patients and their families will be performed at/after T1 in the Intervention group to investigate aspects of behavioral engagement during the use of the Agilik device at home.

Monitoring plan

The investigator undertakes to ensure that the planned procedures and actions comply with the code of good clinical practice - GCP and the applicable regulations.
The Principal Investigator of each clinical site is responsible for data and safety monitoring for this protocol. Patient tolerance with Agilik setup and data collection process will be evaluated during the experimentation. Subjects will be informed that they can ask to stop the experiments at any time.

Enrolment

Each subject is considered enrolled in the study the moment we obtain his/her consent to participate in the research by signing the informed consent to join the research. Recruitment will be carried out at IRCCS Medea, IRCCS Fondazione Stella Maris, Fondazione Don Carlo Gnocchi Onlus Rome, Fondazione Mondino, Centro S. Maria al Mare - Fondazione Don Carlo Gnocchi andFondazione Don Carlo Gnocchi - IRCCS S. Maria Nascente.

Statistical considerations

Data will be analysed using SPSS statistical software. Demographic data and the number of dropouts will be analysed using descriptive statistics. EMG and gait data will be analysed with standardised algorithms. [21,22]
Data collected during the outcome assessments and the follow-up will be compared with T0 data (intra-group analysis). Furthermore, we will compare the results of the Control and Intervention groups (iter-group analysis). Appropriate statistical design will be used according to the normality of the dataset.
Since the Agilik orthosis is purposefully adjusted to increase knee extension as much as possible *above* what was achieved by a standard brace for each subject, we can be certain that the knee flexion will not worsen with it. We will consider an increase in KEMS greater than 8 degrees to be a clinically significant improvement [23]. The trial is designed to find a significant difference for at least one of the two primary multiple endpoints [24]. The standardized effect sizes for 6MWT and KEMS are assumed as 0.4 [14,25] and 1.0 [23,26] and the correlation of the two endpoints is assumed as 0.5 at a significance level of 0.025 with power = 0.80. Sample sizes obtained are n = 18 for each group, with a total sample size required of 2n= 36. Considering potential dropout (10%), we conservatively set the number of total participants at 40. The sample size estimation has been performed in R .

Data management

The indispensable data to finalise the investigation, in particular personal and health data, will be processed in compliance with EU Regulation 2016/679, (GDPR) and Legislative Decree 10 August 2018, no. 101.

During data collection, the PI of each clinical site will pseudonymised the data, identifying the subjects recruited with codes, or adopting other solutions that comply with point 5.4 of the Privacy Guarantor's Order No. 146/2019. Indeed, the use of encryption techniques allows to store and process the subject's information, preventing the identification by any person outside the Institute. Only the PI and authorised personnel can link each code to the name of the participant.
Project partners acting as autonomous data controllers may share information to each other in pseudonymised form and without identification data. Such transmission is configured as data communication and processing by third parties, which must be indicated by name and separately in the information on the processing of personal data.
Subjects who wish could know what information will be stored and in what manner. Access to the data will be allowed only to authorised personnel of the centre involved. At the end of the research, the study results concerning a participant to the investigation may be communicated to him or her, if requested.
All data collected during the course of this project will be kept for a minimum of 25 years past the closing of the protocol.

The above data will be entered in digitalized format, creating a specific eCRF in a Data Management System validated according to national legislation, already in use at IRCCS Medea. The platform used will be RedCap (Research Electronic Data Capture). The REDCap Consortium is made up of more than 1000 institutional partners around the world (research institutions, universities, ministries, etc.). The consortium supports a secure web application (REDCap) designed exclusively to support data capture for research studies. The REDCap application allows users to create and manage online databases quickly and securely, and it is currently in use for more than 110,000 projects with approximately 150,000 users covering numerous areas of research interest across the consortium. Through REDCap, for this study the following measures will be implemented:

- user-level identification, with specific restrictions based on role in the study;
- validation and control of data integrity in real time;
- de-identification of patients before data export;
- centralized data storage with daily backup. The compiler, in turn, will take responsibility for the non-disclosure and dissemination of the data.

Intellectual property

The results of the study will be owned by all the clinical experimental centres that participate in the study.

Information about any amendments to the study

Any changes needed during the study will be appropriately and promptly reported to the Ethics Committee to which these documents were submitted and, if necessary, a specific amendment will be presented.

Policy regarding follow-up and management of any deviations from the protocol

If there will be necessary to make changes to the investigation that could have a significant impact on the safety, health, or rights of the subjects or on the robustness or reliability of the data, the Ethics Committee will be notified within one week. The reasons for such changes will be clearly reported, including an updated version of the relevant documentation. Changes in the relevant documentation

will be clearly identifiable and reported in the section "*Information about any amendments to the CIP*" of this document.
Notification of substantial changes will be accompanied by an opinion of the Ethics Committee requested in advance.

Accountability regarding the device

In the event of a malfunction or any adverse event, researchers will contact the manufacturer and/or the distributor, which will undertake the repair at its own expense.
Contact for device-vigilance: Ing. Paola Grigioni; Email: paola.grigioni@lanostrafamiglia.it

Ethical considerations

The proposed CIP was drafted according to the Regulation EU 2017/754 Annex XV and Chapter VI, UNI EN ISO 14155, standards of Good Clinical Practice of the European Union, in accordance with the Declaration of Helsinki and was approved by the competent Ethics Committee.
All patients will be supervised at all times during the test procedures. All of the PIs with medical or allied health licences (physicians or physical therapists) in this protocol have experience and training in evaluating children with and without movement disorders. Physical therapists will be present for all experimental sessions in the clinical sites.

Description of the Informed consent process

All participants will receive a verbal explanation in terms suited to their level of comprehension of the purposes, procedures and potential risks of the study and of their rights as research participants. Participants' guardians will have the opportunity to carefully review the written consent form and ask questions regarding this study prior to signing. The participants (or their legal tutor) will be provided with an information letter and asked to sign an informed consent form before starting the experimentation.

Safety reporting

Pursuant to EU Regulation 2017/745, MDCG 2020-10/1 Rev1 (Safety reporting in clinical investigation of medical devices under the Regulation EU 2017/745), Legislative Decree 46/97, Legislative Decree 507/92, Meddev Guideline 2.12-1 rev.8 and its supplement "Additional guidance regarding the vigilance system as outlined in MEDDEV 2.12-1 rev.8," all incidents and serious adverse events that may occur during the investigation should be reported to Office V (Medical Device Vigilance) of the DGDFSC of the Ministry of Health and the relevant Ethics Committee. Since this is a clinical study, serious adverse events may also be forwarded to Office VI of the Ministry of Health for information.

Criteria and procedures for follow-up of subjects following the end, temporary halt or early termination of an investigation, for follow-up of subjects who have withdrawn their consent and procedures for subjects lost to follow-up

In case of suspension or early termination of the investigation, the Ethics Committee will be notified. The communication will contain detailed information on the date of suspension or early termination and the related reasons.

Description of the arrangements for taking care of the subjects after their participation in the clinical investigation has ended

Not applicable

Policy for data publication

The results obtained at the end of this clinical trial will be presented at national and international conferences and submitted to peer-reviewed international journals.
Before the study initiation and the recruitment of the first participant, the protocol will be published in clinicaltrials.gov.
The raw data of the study will be published among the supplementary materials of scientific articles and/or uploaded to Zenodo, a multidisciplinary repository, managed by CERN in Geneva, which allows researchers to share and preserve research results in any size and form. Depositing data in ZENODO guarantees their compliance with the FAIR principles.

List of the technical and functional features of the device

Refer to the Instruction for Use attached to the protocol (D0476-18_IFU AGILIK 1.1) for the list of technical and functional features of the device.

Bibliography

1. Molnar GE. Rehabilitation in cerebral palsy. *West J Med*. 1991;154(5):569-572.
2. Yeargin-Allsopp M, Van Naarden Braun K, Doernberg NS, Benedict RE, Kirby RS, Durkin MS. Prevalence of cerebral palsy in 8-year-old children in three areas of the United States in 2002: a multisite collaboration. *Pediatrics*. 2008;121(3):547-554. doi:10.1542/peds.2007-1270
3. Binder H, Eng GD. Rehabilitation management of children with spastic diplegic cerebral palsy. *Arch Phys Med Rehabil*. 1989;70(6):482-489. doi:10.1016/0003-9993(89)90012-9
4. Perry J. Pathologic gait. *Instr Course Lect*. 1990;39:325-331.
5. Rosenbaum PL, Russell DJ, Cadman DT, Gowland C, Jarvis S, Hardy S. Issues in measuring change in motor function in children with cerebral palsy: a special communication. *Phys Ther*. 1990;70(2):125-131. doi:10.1093/ptj/70.2.125
6. Bottos M, Gericke C. Ambulatory capacity in cerebral palsy: prognostic criteria and consequences for intervention. *Dev Med Child Neurol*. 2003;45(11):786-790. doi:10.1017/s0012162203001452
7. Olney SJ, MacPhail HE, Hedden DM, Boyce WF. Work and power in hemiplegic cerebral palsy gait. *Phys Ther*. 1990;70(7):431-438. doi:10.1093/ptj/70.7.431
8. Vuillermin C, Rodda J, Rutz E, Shore BJ, Smith K, Graham HK. Severe crouch gait in spastic diplegia can be prevented: a population-based study. *J Bone Joint Surg Br*. 2011;93(12):1670-1675. doi:10.1302/0301-620X.93B12.27332
9. Damiano DL, Kelly LE, Vaughn CL. Effects of quadriceps femoris muscle strengthening on crouch gait in children with spastic diplegia. *Phys Ther*. 1995;75(8):658-671. doi:10.1093/ptj/75.8.658

10. Wiley ME, Damiano DL. Lower-extremity strength profiles in spastic cerebral palsy. *Dev Med Child Neurol*. 1998;40(2):100-107. doi:10.1111/j.1469-8749.1998.tb15369.x

11. Rogozinski BM, Davids JR, Davis RB 3rd, Jameson GG, Blackhurst DW. The efficacy of the floor-reaction ankle-foot orthosis in children with cerebral palsy. *J Bone Joint Surg Am*. 2009;91(10):2440-2447. doi:10.2106/JBJS.H.00965

12. Shideler BL, Bulea TC, Chen J, Stanley CJ, Gravunder AJ, Damiano DL. Toward a hybrid exoskeleton for crouch gait in children with cerebral palsy: neuromuscular electrical stimulation for improved knee extension. *J Neuroeng Rehabil*. 2020;17(1):121. doi:10.1186/s12984-020-00738-7

13. Damiano DL, DeJong SL. A systematic review of the effectiveness of treadmill training and body weight support in pediatric rehabilitation. *J Neurol Phys Ther*. 2009;33(1):27-44. doi:10.1097/NPT.0b013e31819800e2

14. Beretta E, Storm FA, Strazzer S, et al. Effect of Robot-Assisted Gait Training in a Large Population of Children With Motor Impairment Due to Cerebral Palsy or Acquired Brain Injury. *Arch Phys Med Rehabil*. 2020;101(1):106-112. doi:10.1016/j.apmr.2019.08.479

15. Lerner ZF, Damiano DL, Bulea TC. The Effects of Exoskeleton Assisted Knee Extension on Lower-Extremity Gait Kinematics, Kinetics, and Muscle Activity in Children with Cerebral Palsy. *Sci Rep.* 2017 Oct 18;7(1):13512. doi: 10.1038/s41598-017-13554-2. PMID: 29044202; PMCID: PMC5647342.

16. Kim SK, Park D, Yoo B, Shim D, Choi JO, Choi TY, Park ES. Overground Robot-Assisted Gait Training for Pediatric Cerebral Palsy. *Sensors* (Basel). 2021 Mar 16;21(6):2087. doi: 10.3390/s21062087. PMID: 33809758; PMCID: PMC8002375.

17. Dierwechter B, Kolakowsky-Hayner SA. Journey to 1 Million Steps: A Retrospective Case Series Analyzing the Implementation of Robotic-Assisted Gait Training Into an Outpatient Pediatric Clinic. *Pediatr Phys Ther.* 2024 Feb 14. doi: 10.1097/PEP.0000000000001097. Epub ahead of print. PMID: 38349640.

18. Smania N, Gandolfi M, Marconi V, et al. Applicability of a new robotic walking aid in a patient with cerebral palsy. Case report. *Eur J Phys Rehabil Med*. 2012;48(1):147-153

19. Hunt M, Everaert L, Brown M, Muraru L, Hatzidimitriadou E, Desloovere K. Effectiveness of robotic exoskeletons for improving gait in children with cerebral palsy: A systematic review. *Gait Posture*. 2022 Oct;98:343-354. doi: 10.1016/j.gaitpost.2022.09.082. Epub 2022 Sep 26. PMID: 36306544.

20. Harris PA, Taylor R, Thielke R, Payne J, Gonzalez N, Conde JG. Research electronic data capture (REDCap)—A metadata-driven methodology and workflow process for providing translational research informatics support. *J Biomed Inform*. 2009;42(2):377-381. doi:https://doi.org/10.1016/j.jbi.2008.08.010

21. Peri E, Panzeri D, Beretta E, Reni G, Strazzer S, Biffi E. Motor Improvement in Adolescents Affected by Ataxia Secondary to Acquired Brain Injury: A Pilot Study. *Biomed Res Int*. 2019;2019:8967138. doi:10.1155/2019/8967138

22. Biffi E, Beretta E, Storm FA, et al. The Effectiveness of Robot- vs. Virtual Reality-Based Gait Rehabilitation: A Propensity Score Matched Cohort. *Life (Basel, Switzerland)*. 2021;11(6). doi:10.3390/life11060548

23. Daly JJ, Wolpaw JR. Brain-computer interfaces in neurological rehabilitation. *Lancet Neurol*. 2008;7(11):1032-1043. doi:10.1016/S1474-4422(08)70223-0

24. Sozu T, Sugimoto T, Hamasaki T, Evans SR. Sample Size Determination in Clinical Trials with Multiple Endpoints. ISBN 978-3-319-22005-5. doi:10.1007/978-3-319-22005-5

25. Storm FA, Petrarca M, Beretta E, et al. Minimum Clinically Important Difference of Gross Motor Function and Gait Endurance in Children with Motor Impairment : A Comparison of Distribution-Based Approaches. *Biomed Res Int.* 2020;2020:2794036. doi:10.1155/2020/2794036

26. Bonnefoy-Mazure A, Sagawa Y, Lascombes P., De Coulon G., Armand S. Identification of gait patterns in individuals with cerebral palsy using multiple correspondence analysis, *Res. Dev. Disabil*. 2013;34(9):2684-2693. doi:10.1016/j.ridd.2013.05.002
